# Exclusion criteria in clinical trials of treatments for neuropathic pain: a systematic analysis

**DOI:** 10.3389/fpain.2026.1716686

**Published:** 2026-03-18

**Authors:** Raymundo Salcedo, Reihaneh Moghadam, Sahar Saneifard, Alexander Zhou, Vafi Salmasi

**Affiliations:** 1 Chicago Medical School, Rosalind Franklin University of Medicine and Science, Chicago, IL, United States; 2 Department of Medical Education, University of Southern California, Keck School of Medicine, Los Angeles, CA, United States; 3 School of Engineering, University of California Berkeley, Berkeley, CA, United States; 4 Department of Cognitive Science, University of California San Diego, San Diego, CA, United States; 5 Department of Anesthesiology, Perioperative and Pain Medicine, Stanford University School of Medicine, Stanford, CA, United States

**Keywords:** eligibility criteria, exclusion, external validity, generalizability, neuropathic pain

## Abstract

**Introduction:**

Clinical trials for neuropathic pain often employ strict exclusion criteria that may limit the generalizability of their findings to real-world clinical populations. This study systematically analyzed the nature, prevalence, and reporting quality of exclusion criteria in neuropathic pain trials.

**Methods:**

We conducted a systematic review of studies published from 2012 to 2022 to analyze exclusion criteria from clinical trials studying treatments for neuropathic pain. We extracted data on the number, type, and frequency of exclusion criteria used. We also analyzed patient flow metrics, including screening, eligibility, enrollment, and completion rates, identified key missing information, and performed correlations between different exclusion criteria to establish patterns in exclusion criteria use.

**Results:**

We included 161primary clinical trial publications of neuropathic pain interventions in our analysis. Most trials examined medication-based interventions and were placebo/sham controlled. The median number of exclusion criteria per study was 5 (IQR 4–7)). Medical comorbidities (86.4%), age restrictions (71.0%), and minimum pain score requirements (71.6%) were used most often as exclusion criteria. Psychological comorbidities were excluded in 56.8% of trials, despite being common in chronic pain populations. Only 36.4% of trials reported the number of patients screened, and 43.8% reported eligibility numbers, highlighting significant gaps in transparent reporting. Among trials that did report patient flow metrics, the mean eligibility rate was 67.9% of screened patients, while the mean enrollment rate was 60.9% of screened patients. We observed moderate correlations between certain exclusion criteria, particularly between minimum pain duration and score requirements (*r* = 0.56), and weak correlation between the presence of other painful conditions and patients on other treatments (*r* = 0.40).

**Conclusions:**

Our findings demonstrate that neuropathic pain trials frequently employ multiple exclusion criteria that may significantly limit their generalizability to clinical practice. The high prevalence of psychological comorbidity exclusions is particularly concerning given their common co-occurrence with chronic pain. Additionally, inconsistent reporting of patient flow metrics hampers the assessment of how exclusion criteria affect trial recruitment and generalizability. We recommend standardization of exclusion criteria reporting and careful consideration of whether strict exclusions truly serve trial objectives.

**Systematic Review Registration:**

https://www.crd.york.ac.uk/PROSPERO/view/CRD42023387885, identifier CRD42023387885.

## Introduction

Neuropathic pain, the pain resulting from a lesion or disease within the somatosensory nervous system [(1). International Association for the Study of Pain (IASP). Retrieved April 4, 2025, n.d.], affects approximately 7%–10% of the general population (2, 3) and represents a significant challenge in pain management. Neuropathic pain occurs in various conditions, including diabetic peripheral neuropathy, postherpetic neuralgia and trigeminal neuralgia (4, 5). While randomized controlled trials (RCTs) are considered the gold standard for evaluating treatment efficacy, they are typically designed to prioritize internal validity—ensuring safety, mechanistic clarity, and statistical power within narrowly defined populations—rather than broad generalizability. Accordingly, exclusion criteria are often necessary and appropriate, particularly in early-phase efficacy trials or studies targeting specific neuropathic pain pathologies. Importantly, individual trials generally do not claim applicability beyond their enrolled populations. However, when considered collectively, consistent patterns of restrictive eligibility criteria across trials may create a structural mismatch between studied populations and the patients most commonly encountered in clinical practice. This issue is particularly relevant in neuropathic pain, where multimorbidity, psychological comorbidities, and concurrent therapies are common. Thus, the present review does not critique individual trial design choices, but rather examines how the aggregate use of exclusion criteria across the literature may limit the evidentiary base available for managing complex, real-world patients (6–9).

Previous research has highlighted concerns about the external validity of RCTs across various medical disciplines (10–12). However, researchers have paid limited attention to this issue in neuropathic pain trials, with analyses addressing external validity and generalizability concerns by examining such factors as the relationship between study outcomes and the frequency of publication citations (13) the lack of functional outcome measures (14) in trial designs, or exclusion of a single comorbidity, e.g., depression (15). Our group is specifically interested in exclusion patterns in randomized clinical trials for treatment of chronic pain. Building on previous work investigating the impacts of exclusion criteria on the external validity of neurological disorders (16, 17) and our own analyses of chronic pain trials (18), we sought to continue our series by applying the same approach to use of exclusion criteria in neuropathic pain studies. Compared to other chronic pain conditions, neuropathic pain has relatively more defined pathology and diagnostic criteria. We therefore conducted a systematic review of exclusion criteria in neuropathic pain trials. Understanding the nature and impact of exclusion criteria is crucial for interpreting trial results and applying them appropriately in clinical practice.

## Methods

### Protocol registration

The systematic review protocol was registered with PROSPERO (CRD42023387885) prior to conducting the search.

### Search strategy and study selection

We conducted a systematic search of PubMed/MEDLINE database while filtering for only clinical trials published between January 1, 2012 and December 31, 2022. The complete search string was:

(pain[ti] OR painful[ti]) AND (neuropathic[ti] OR neuropathy[ti] OR neuralgia[ti] OR neuritis[ti] OR radiculopathy[ti] OR radiculitis[ti] OR plexopathy[ti] OR radicular[ti]).

Two authors (VS and RS) independently reviewed all identified articles. Disagreements were resolved through discussion between all authors until unanimous agreement could be reached.

For this systematic review, studies were included if they: 1) were clinical trials of neuropathic pain interventions published between January 1, 2012 and December 31, 2022; 2) reported outcomes related to pain or function; and 3) were primary reports (i.e., not secondary analyses or validation studies). Studies were excluded if they: 1) combined data from multiple trials for dose titration analyses, 2) reported no pain or functional outcomes, 3) were secondary analyses of previously published trials, or 4) validated screening tools for neuropathic pain.

### Data collection

For each eligible trial, we extracted 1) study characteristics (year of publication, type of intervention, control group type, sample size), 2) recruitment metrics (number of patients screened, number eligible, number enrolled, number completed, number declining participation), and 3) exclusion criteria (medical comorbidities, psychological conditions, age restrictions, pain characteristics, other eligibility criteria).

To contextualize exclusion criteria relative to study intent, trials were categorized by intervention type (pharmacologic vs. procedural) and by therapeutic intent (primary efficacy trials of novel agents vs. trials evaluating previously approved therapies for new indications). Although many medication-based studies were primary efficacy trials requiring pathology-specific eligibility criteria, all eligible trials were retained to evaluate aggregate exclusion patterns across the neuropathic pain literature. A full list of included studies with intervention type is provided in Supplementary Appendix.

### Statistical analysis

Data analysis was performed using Python 3.8 with pandas, numpy, and seaborn libraries. For descriptive statistical analyses, we calculated frequencies and percentages for categorical variables, mean, median, and range for continuous variables, and conducted a distribution analysis of exclusion criteria per study. Correlation analyses were performed using Pearson correlation coefficients between different exclusion criteria and visualization using heatmaps to identify patterns of co-occurrence.

Patient flow metrics involved the calculation of key patient ratios: eligible/screened (%), enrolled/screened (%), completed/screened (%), completed/enrolled (%), enrolled/eligible (%), and completed/eligible (%). Additionally, we analyzed missing data patterns in reporting. Temporal trend analysis was performed using a year-by-year analysis of publication. Missing data was handled through complete case analysis for each metric.

## Results

### Search results and treatment/control methodologies

**Figure 1:**
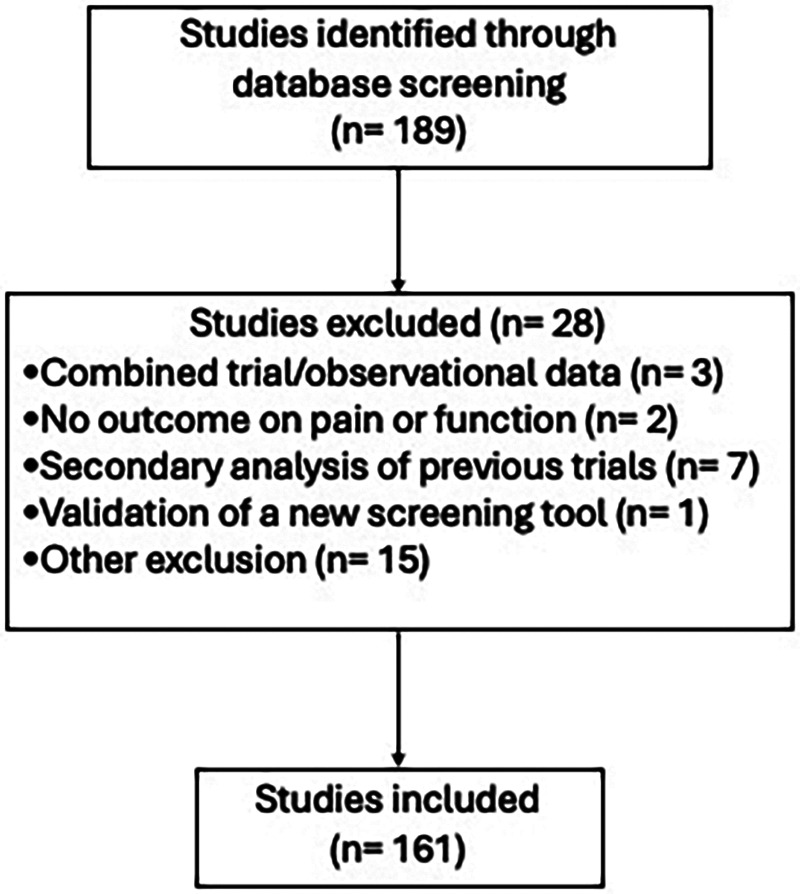
Study flow chart showing the systematic review process. From 189 initially identified studies, 28 were excluded based on specified criteria, resulting in 161 studies included in the final analysis.

Our initial search found 189 papers, but we excluded 28 papers as below (Figure 1), leaving 161 papers for analysis:

Four papers (19–22)(1, 2, 3) reported combined data from the treatment arms of multiple clinical trials and an observational study to assess dose titration. There were studies that did not report any outcomes related to pain or function; we thus excluded these studies from our analysis (23–27).

We also excluded papers that reported either protocol or secondary analysis of otherwise published clinical trial with similar eligibility criteria (21 papers).

Among the 161 neuropathic pain clinical trial publications we included in our analysis, the number per year grew considerably from 2012 to 2022 (Table 1). While only one paper was published in 2012, we found an average of 17 papers published per year from 2013 to 2021.

**Table 1:** Exclusion criteria frequency by intervention type (pharmacologic vs. procedural).

Exclusion Criterion	Pharmacologic (*N* = 95)	Pharmacologic (%)	Procedural (*N* = 12)	Procedural (%)
Medical comorbidity	86	90.53%	11	91.67%
Minimum Pain Score	78	82.11%	9	75.00%
Age Range	74	77.89%	9	75.00%
Minimum Pain Duration	65	68.42%	8	66.67%
Patients on Other Treatment(s)	64	67.37%	5	41.67%
Presence of Other Painful Condition(s)	56	58.95%	6	50.00%
Psychological comorbidity	54	56.84%	9	75.00%
Substance Use	33	34.74%	3	25.00%
Language Barrier	4	4.21%	5	41.67%
Pending Litigation	3	3.16%	2	16.67%

The majority of trials studied medication-based interventions (95 studies), followed by pain procedures (11 studies), surgical procedures, physical therapy, behavioral therapy and other alternative treatment modalities (e.g., acupuncture, transcranial magnetic stimulation, etc.). In distribution of control type placebo/sham control studies were most common (106 studies), followed by routine care studies (27 studies); five studies did not have any controls (Table 1).

### Number and frequency of exclusion criteria

**Figure 2:**
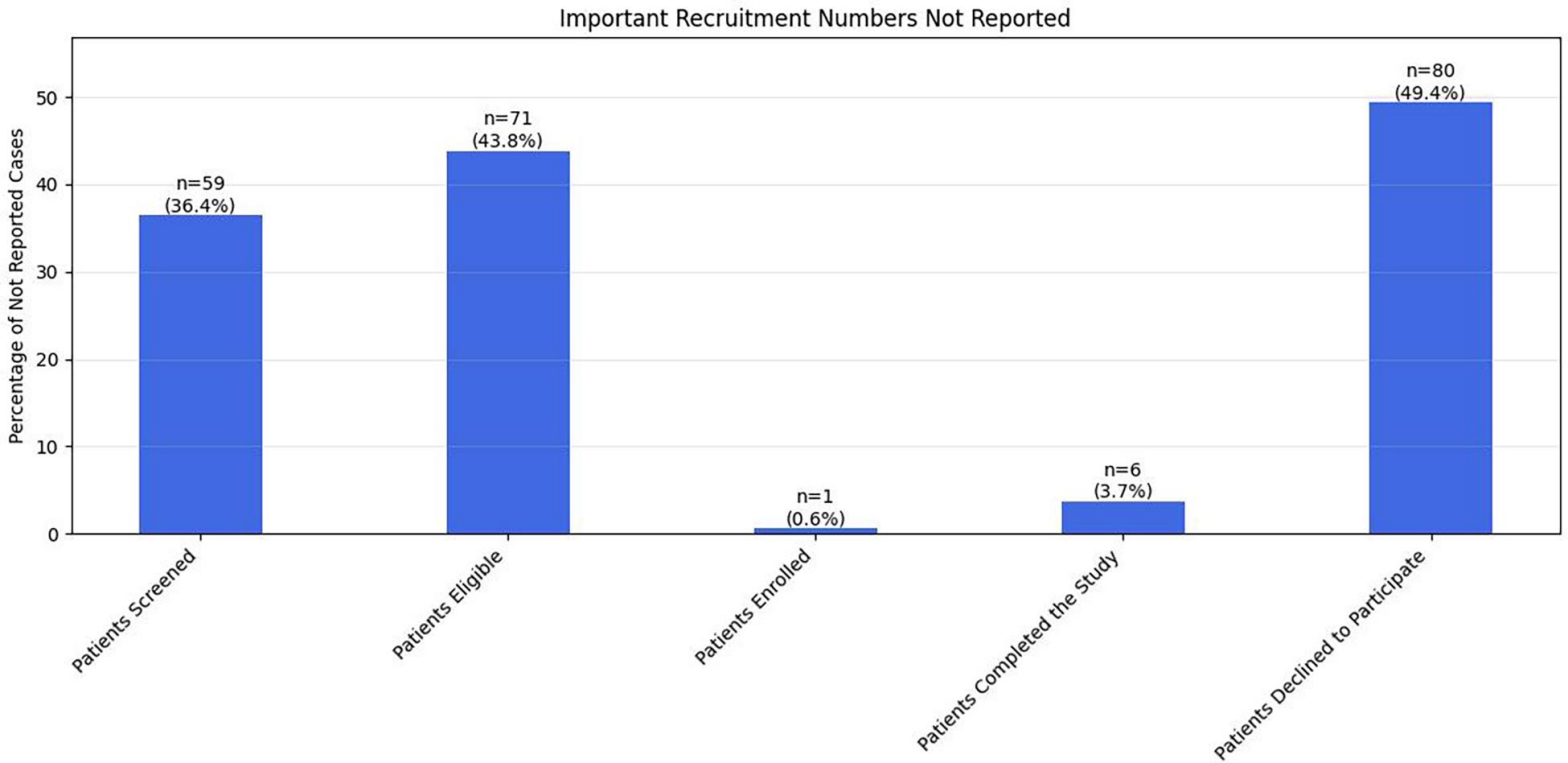
Distribution of recruitment conversion rates across studies. Six histograms display the frequency distributions of various recruitment rate metrics with mean and median values indicated by dashed lines. The distributions show: eligible/screened rates (top left, mean 67.9%, median 71.7%), enrolled/screened rates (top middle, mean 60.9%, median 65.1%), enrolled/eligible rates (top right, mean 86.9%, median 95.4%), completed/screened rates (bottom left, mean 53.0%, median 55.3%), completed/eligible rates (bottom middle, mean 78.0%, median 84.4%), and completed/enrolled rates (bottom right, mean 88.7%, median 93.5%). Most distributions show right-skewed patterns with higher completion rates at later stages of the recruitment funnel.

Across 161 studies, the median number of exclusion criteria per study was 5 (IQR 4–7). The vast majority (*n* = 136; 84.4%) of studies used 4–9 exclusion criteria; 23 (14.2%)studies used 3 or fewer exclusion criteria, and only 3 (1.8%) studies used greater than 10 exclusion criteria. Figure 2 summarizes the important eligibility criteria not reported in different studies.

**Figure 3:**
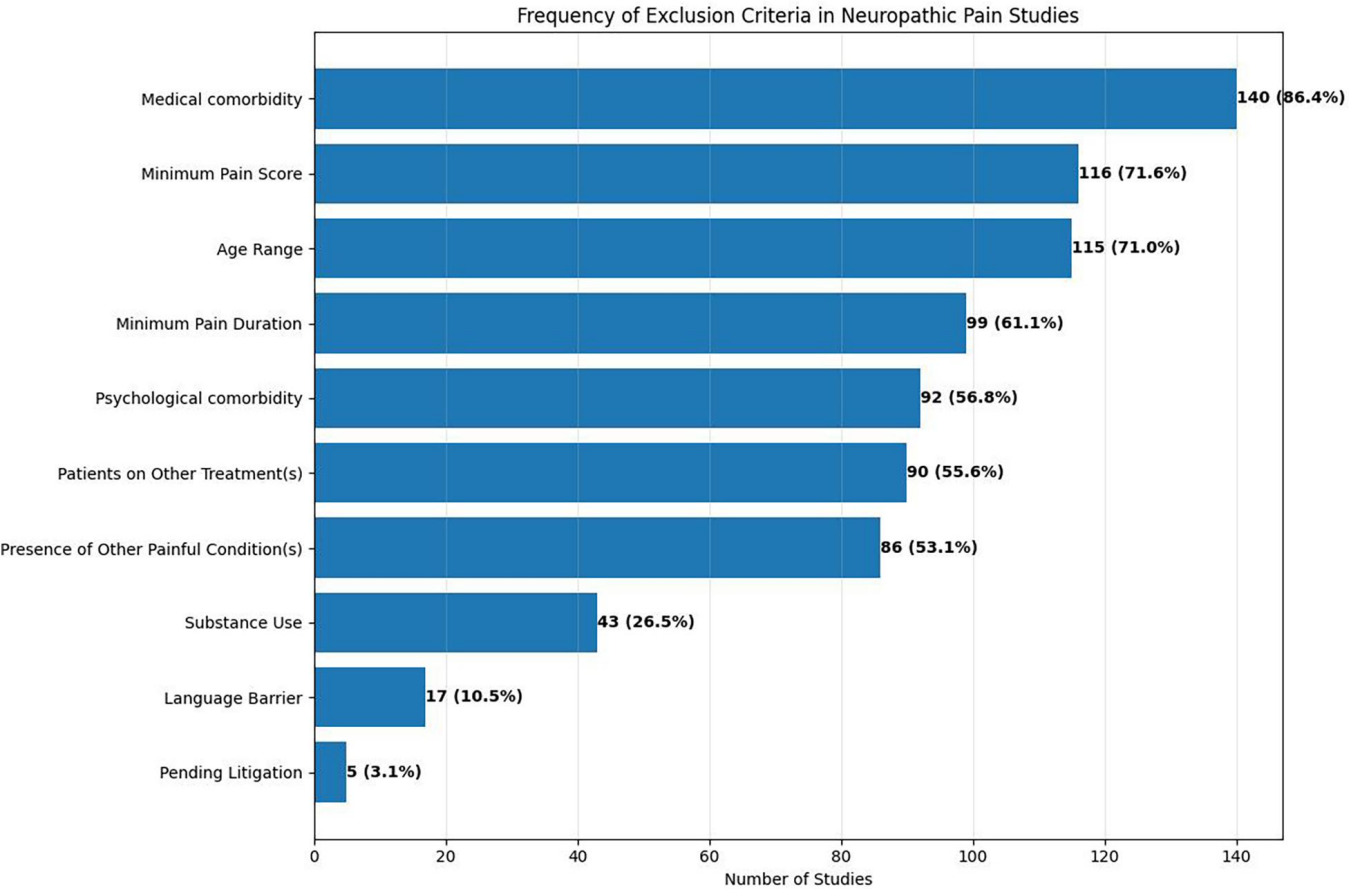
Frequency of exclusion criteria in neuropathic pain studies. The horizontal bar chart displays the most commonly used exclusion criteria across 162 studies, with both absolute numbers and percentages shown. Medical comorbidity was the most frequent exclusion criterion (140 studies, 86.4%), followed by minimum pain score requirements (116 studies, 71.6%) and age restrictions (115 studies, 71.0%). Less common criteria included substance use (43 studies, 26.5%), language barriers (17 studies, 10.5%), and pending litigation (5 studies, 3.1%).

Over 70% of trials excluded participants with other comorbid medical conditions (86.4%), those who did not meet minimum pain score requirements (71.6%), and those who did not fall within specific age ranges (71.0%) (Figure 3). Approximately 61% of trials required pain to persist for a minimum duration. Additionally, over half the trials excluded participants with psychological comorbidities (56.8%), patients on other treatments (55.6%), and those who had another painful condition (53.1%). Fewer than 30% of trials excluded patients using substances, had a language barrier, or were involved in pending litigation.

When stratified by intervention type, pharmacologic trials employed a higher median number of exclusion criteria than procedural trials (median 6 vs. 4). Pharmacologic trials more frequently excluded participants with medical comorbidities (89.5% vs. 63.6%) and psychological comorbidities (60.0% vs. 36.4%). In contrast, procedural trials more commonly excluded participants based on prior interventions or anatomic considerations. These findings were not statistically tested but suggest that trial purpose and intervention modality meaningfully shape exclusion practices.

### Patient flow metrics

**Figure 4:**
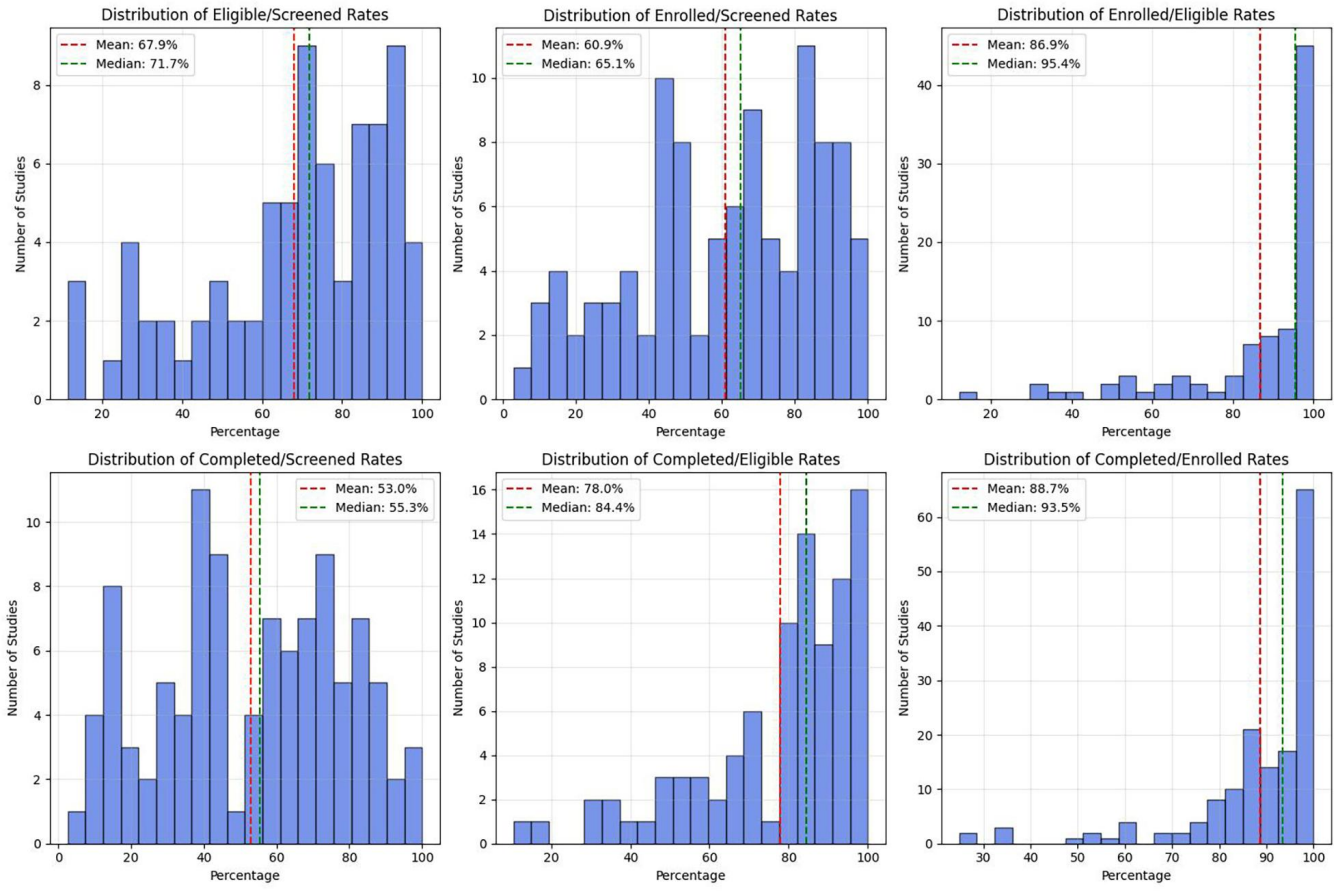
Distribution of the total number of exclusion criteria per study. The histogram shows that most neuropathic pain studies employed 5–7 exclusion criteria, with the peak at 6 criteria (29 studies). The distribution is roughly normal, ranging from 0 to 11 exclusion criteria per study, with very few studies using either very few (0–1) or very many (10−11) exclusion criteria.

We also examined patient flow metrics from these studies, and found that several important recruitment numbers were frequently unreported (Figure 4) Approximately a third to one-half of trials examined did not report the number of patients screened (36.4%), the number of eligible patients (43.8%), and the number of patients declining to participate (49.4%), while nearly all trials reported the numbers of enrolled patients and patients completing the study. In the trials that did report these metrics approximately two-thirds (mean, 67.9%) of those screened met the eligibility criteria, and of those eligible, a high percentage enrolled (mean, 86.9%), which corresponded to a mean of 60.9% of those screened. Nearly 89% of enrolled participants completed the studies, indicating a high retention rate, which comprised 78% of those eligible, and 55.3% of those initially screened.

### Correlations between exclusion criteria

To understand the patterns underlying the application of exclusion criteria, we performed correlation analyses (Appendix Figure S1). We found a notable correlation between the minimum pain duration and minimum pain score (*r* = 0.56), suggesting that trials requiring a longer pain duration were more likely to require a higher minimum pain score. Additionally, we observed moderate positive correlations between other exclusion criteria, including patients with other painful conditions/patients on other treatments (*r* = 0.40), patients with medical comorbidities/psychological comorbidities (*r* = 0.34), patients on other treatments/minimum pain score (*r* = 0.34), and patients on other treatments/minimum pain duration (*r* = 0.33). Overall, the correlation analyses indicated that use of specific exclusion criteria across studies appeared to be fairly independent, with no criterion strongly predicting use of another.

## Discussion

Our findings highlight several concerning trends in neuropathic pain trials:

First, the high prevalence of exclusion criteria related to medical and psychological comorbidities or relating to patients with multiple painful conditions or those undergoing treatment suggests that trial populations may poorly represent real-world patients, who often present with multiple comorbidities, comorbid pain conditions, or with multi-modal or combination treatments.

Over 70% of trials (86.4%) excluded participants with comorbid medical conditions, a strategy aimed at enhancing sample homogeneity and reducing potential confounding. However, this approach limits the external validity of the findings, as comorbidities are common among individuals with chronic pain and often influence treatment outcomes in real-world clinical settings.

Participants who did not meet minimum pain score requirements were excluded in 71.6% of trials. While this ensures assessment in individuals experiencing clinically significant pain, it may exclude patients with mild to moderate pain who could nonetheless benefit from interventions. This highlights how eligibility criteria influence the patient population and potential applicability of findings.

Approximately 61% of trials required pain to persist for a minimum duration, a criterion that supports the inclusion of individuals with chronic pain and enhances the specificity of the studied condition. However, this requirement may inadvertently exclude patients with emerging or subacute pain, thereby limiting the applicability of findings to earlier stages of pain development and potentially delaying opportunities for early intervention.

Over half of the trials (56.8%) excluded participants with psychological comorbidities. While this approach may improve internal validity by minimizing confounding from mental health factors, it raises concerns regarding external validity. Psychological conditions such as anxiety and depression are well-documented to influence pain perception and are prevalent among individuals with chronic pain. As such, the exclusion of these patients may limit the generalizability of trial findings to routine clinical populations, where psychological comorbidities are common (28–30). Patients receiving other treatments were excluded in 55.6% of trials, a measure intended to prevent potential interference with the study intervention and preserve internal validity. However, this exclusion criterion may reduce the generalizability of trial findings, as pain management in real-world settings often involves multiple concurrent therapies rather than a single intervention (31).

Second, inconsistent reporting of patient flow metrics limits the ability to assess how exclusion criteria influence trial reach and representativeness. Among trials reporting screening outcomes, nearly one-third of screened patients failed eligibility criteria. While such attrition is expected in efficacy-driven trials, its cumulative effect across the literature disproportionately excludes patients with greater clinical complexity. Notably, once enrolled, participants demonstrated high retention (89% completion), suggesting that trial procedures and interventions were generally acceptable. This contrast highlights an important opportunity for future research: designing complementary trials that selectively relax exclusion criteria to assess treatment effects in patients with comorbidities, concurrent therapies, or mixed pain phenotypes. Without such studies, the evidence base remains weighted toward less complex patients, limiting applicability to real-world clinical populations.

The incomplete reporting of patient flow data represents a critical missed opportunity to understand recruitment efficiency and population representativeness. Approximately two-thirds (67.9%) of screened individuals met eligibility criteria, meaning nearly one-third were excluded before enrollment. While this exclusion rate may not appear extreme, it masks important recruitment dynamics: the screening-to-completion cascade shows that only 55.3% of initially screened patients ultimately finished the trial. This attrition pattern—where one-third are excluded at screening and another segment lost between enrollment and completion—compounds the restriction of the study sample. The high post-enrollment retention (89% of enrolled participants completing) demonstrates that once patients enter trials, adherence is strong, suggesting that the interventions and procedures themselves are not primary barriers to participation.

This discrepancy between screening-stage exclusion and post-enrollment retention reveals a fundamental tension in current trial design. The exclusion of clinically complex patients occurs predominantly at screening, yet these same patients might tolerate trial procedures well if enrolled. This pattern suggests that many exclusion criteria function more to preserve internal validity than to protect participants or ensure feasibility. The result is a systematic enrichment of the evidence base with data from less complex patients, while knowledge gaps persist for those with comorbidities, polypharmacy, or atypical pain presentations—precisely the patients most commonly encountered in clinical practice.

Furthermore, the failure to report key recruitment metrics such as screening numbers, reasons for ineligibility, and declination rates limits assessment of trial reach and potential selection bias. Without transparency about who was approached, who was excluded and why, and who declined participation, the representativeness of enrolled samples remains uncertain. This opacity also obscures whether certain patient subgroups face systematic barriers to trial participation, whether through overly restrictive eligibility criteria, logistical challenges, or design features that inadvertently favor less complex patients. Such underreporting violates CONSORT guidelines and compromises the interpretability and reproducibility of trial findings, making it difficult for future investigators to design more inclusive studies or for clinicians to assess the applicability of results to their patient populations.

Third, even though individual trials are appropriately designed to address specific clinical questions and generally do not advocate for application beyond their test populations, there were patterns of moderately correlated exclusion criteria suggesting certain patient subgroups are systematically underrepresented across studies.Notably, excluding patients who had both a low pain score and low pain duration might overemphasize patients with chronic and severe pain. This would exclude patients whose disease process has not yet progressed to that severe state and potentially hinder research that prevents the progression from acute to chronic pain.

We found a notable positive correlation between the minimum pain duration and minimum pain score required for eligibility (*r* = 0.56), suggesting that trials requiring a longer history of pain were also more likely to set higher thresholds for pain severity. This pattern implies a predominant focus on chronic and severe pain populations, which, while methodologically sound for studying established pain conditions, may inadvertently exclude individuals with moderate symptoms or early-stage pain. Such exclusion risks overlooking opportunities for early intervention, which is crucial for preventing the progression from acute to chronic pain. By prioritizing more advanced pain presentations, trial designs may unintentionally introduce bias that limits both preventive research and the development of interventions applicable to a broader range of clinical scenarios.

We also observed moderate positive correlations between certain exclusion criteria, such as the exclusion of patients with other painful conditions and those receiving other treatments (*r* = 0.40). This suggests a preference for enrolling pain-naive or single-condition populations, likely aimed at reducing confounding variables and enhancing internal validity. However, this approach is not reflective of real-world pain populations, where comorbid pain conditions and the use of multimodal treatment strategies are common. By selecting for highly controlled and homogeneous samples, these trials may not capture the complexity of pain management as it occurs in clinical practice, potentially limiting the generalizability and applicability of trial findings to typical patients (32, 33).

We observed a moderate positive correlation between trials that excluded participants with medical comorbidities and those that excluded individuals with psychological comorbidities (*r* = 0.34). This pattern suggests a tendency toward selecting highly controlled, homogenous populations—likely to enhance internal validity. However, excluding patients with psychological disorders, particularly in the context of chronic pain, raises significant concerns about external validity. There is a well-documented bidirectional relationship between chronic pain and mental health conditions such as anxiety and depression as described above.

We observed a moderate positive correlation between trials that excluded participants receiving other treatments and those that applied higher minimum pain score thresholds (*r* = 0.34). This suggests that studies requiring higher baseline pain severity may also preferentially enroll participants who are not currently managing their pain with existing therapies, potentially in an effort to isolate treatment effects and preserve internal validity. However, this approach raises concerns regarding real-world applicability, as many individuals with chronic pain are already engaged in multimodal or combination therapy regimens. By systematically excluding those receiving concurrent treatments, these trials may underrepresent the typical clinical population and overlook how new interventions perform in the context of ongoing pain management strategies. As such, findings may be less generalizable to real-world settings where polytherapy is the norm, not the exception (18).

### Limitations

This study has several limitations that warrant consideration. First, our analysis was limited to published randomized controlled trials indexed in PubMed/MEDLINE between 2012 and 2022, which may have excluded relevant trials from other databases or unpublished studies, potentially introducing publication bias. Second, although we aimed to comprehensively capture exclusion criteria, some trials lacked consistent or detailed reporting of patient flow metrics and eligibility details, which may have impacted the accuracy of our frequency and correlation analyses. Third, while we quantified exclusion criteria and analyzed their co-occurrence, we did not assess how these criteria influenced trial outcomes, treatment efficacy, or safety profiles. Furthermore, our review did not explore the rationale authors used when selecting specific exclusion criteria, nor did it evaluate how inclusion/exclusion impacted subgroups such as older adults, racially diverse populations, or those with limited access to care. Finally, while we conducted correlation analyses, these were exploratory and do not imply causation. Future studies should investigate how exclusion criteria affect treatment generalizability, outcomes, and healthcare equity, particularly in real-world, comorbid populations commonly seen in pain management settings.

## Conclusion

Neuropathic pain trials appropriately employ exclusion criteria to meet specific scientific objectives. However, when considered collectively, current eligibility practices may limit the applicability of trial findings to the complex patients most commonly encountered in clinical care. Inconsistent reporting of patient flow metrics further obscures the impact of exclusion criteria on trial reach. Pairing traditional efficacy trials with more inclusive and pragmatic designs may strengthen the evidence base for managing neuropathic pain in real-world settings.

## Data Availability

The data analyzed in this study is subject to the following licenses/restrictions: The entirety of the data collected and extracted for systematic review will be available upon appropriate request. Requests to access these datasets should be directed to Vafi Salmasi (vsalmasi@stanford.edu).
