# Supplementary material for: Exclusion criteria in clinical trials of treatments for neuropathic pain: a systematic analysis
Source: Front Pain Res (Lausanne). 2026 Mar 18;7:1716686. doi: 10.3389/fpain.2026.1716686 (PMC13038933; doi:10.3389/fpain.2026.1716686)
Supplement: Supplementary file 1 [file Table1.docx]

**Appendix**

1. Abbas, A. M., Wagdy, W. M., Salem, M. N., & Abdelqader, A. M. (2018). Effect of oral diclofenac potassium plus cervical lidocaine cream on pain perception during hysterosalpingography: A randomized, double-blind, placebo-controlled trial. *Middle East Fertility Society Journal*, *23*(1), 52–56.<https://doi.org/10.1016/j.mefs.2017.06.004>
2. Abdallah, K., Monconduit, L., Artola, A., Luccarini, P., & Dallel, R. (2015). GABAAergic inhibition or dopamine denervation of the A11 hypothalamic nucleus induces trigeminal analgesia. *Pain*, *156*(4), 644–655.<https://doi.org/10.1097/j.pain.0000000000000091>
3. Abu-Shennar, J. A., & Bayraktar, N. (2022). The Effect of Educational Program on Pain Management, Self-Efficacy Behavior, and Quality of Life among Adult Diabetic Patients with Peripheral Neuropathy Pain: A Randomized Controlled Trial. *Experimental and Clinical Endocrinology & Diabetes: Official Journal, German Society of Endocrinology [and] German Diabetes Association*, *130*(8), 509–518.<https://doi.org/10.1055/a-1561-8392>
4. Agarwal, M. M., & Elsi Sy, M. (2017). Gabapentenoids in pain management in urological chronic pelvic pain syndrome: Gabapentin or pregabalin? *Neurourology and Urodynamics*, *36*(8), 2028–2033.<https://doi.org/10.1002/nau.23225>
5. Ahmed, S. U., Zhang, Y., Chen, L., Cohen, A., St Hillary, K., Vo, T., Houghton, M., & Mao, J. (2015). Effect of 1.5% Topical Diclofenac on Clinical Neuropathic Pain. *Anesthesiology*, *123*(1), 191–198.<https://doi.org/10.1097/ALN.0000000000000693>
6. Allison, D. J., Thomas, A., Beaudry, K., & Ditor, D. S. (2016). Targeting inflammation as a treatment modality for neuropathic pain in spinal cord injury: A randomized clinical trial. *Journal of Neuroinflammation*, *13*(1), 152.<https://doi.org/10.1186/s12974-016-0625-4>
7. Andrade, S. M., de Brito Aranha, R. E. L., de Oliveira, E. A., de Mendonça, C. T. P. L., Martins, W. K. N., Alves, N. T., & Fernández-Calvo, B. (2017). Transcranial direct current stimulation over the primary motor vs prefrontal cortex in refractory chronic migraine: A pilot randomized controlled trial. *Journal of the Neurological Sciences*, *378*, 225–232.<https://doi.org/10.1016/j.jns.2017.05.007>
8. Andresen, S. R., Bing, J., Hansen, R. M., Biering-Sørensen, F., Johannesen, I. L., Hagen, E. M., Rice, A. S. C., Nielsen, J. F., Bach, F. W., & Finnerup, N. B. (2016). Ultramicronized palmitoylethanolamide in spinal cord injury neuropathic pain: A randomized, double-blind, placebo-controlled trial. *Pain*, *157*(9), 2097–2103.<https://doi.org/10.1097/j.pain.0000000000000623>
9. Atalay, N. S., Sahin, F., Atalay, A., & Akkaya, N. (2013). Comparison of efficacy of neural therapy and physical therapy in chronic low back pain. *African Journal of Traditional, Complementary, and Alternative Medicines: AJTCAM*, *10*(3), 431–435.<https://doi.org/10.4314/ajtcam.v10i3.8>
10. Attal, N., Ayache, S. S., Ciampi De Andrade, D., Mhalla, A., Baudic, S., Jazat, F., Ahdab, R., Neves, D. O., Sorel, M., Lefaucheur, J.-P., & Bouhassira, D. (2016). Repetitive transcranial magnetic stimulation and transcranial direct-current stimulation in neuropathic pain due to radiculopathy: A randomized sham-controlled comparative study. *Pain*, *157*(6), 1224–1231.<https://doi.org/10.1097/j.pain.0000000000000510>
11. Attal, N., & Bouhassira, D. (2021). Advances in the treatment of neuropathic pain. *Current Opinion in Neurology*, *34*(5), 631–637.<https://doi.org/10.1097/WCO.0000000000000980>
12. Attal, N., de Andrade, D. C., Adam, F., Ranoux, D., Teixeira, M. J., Galhardoni, R., Raicher, I., Üçeyler, N., Sommer, C., & Bouhassira, D. (2016). Safety and efficacy of repeated injections of botulinum toxin A in peripheral neuropathic pain (BOTNEP): A randomised, double-blind, placebo-controlled trial. *The Lancet. Neurology*, *15*(6), 555–565.<https://doi.org/10.1016/S1474-4422(16)00017-X>
13. Baba, M., Matsui, N., Kuroha, M., Wasaki, Y., & Ohwada, S. (2019). Mirogabalin for the treatment of diabetic peripheral neuropathic pain: A randomized, double-blind, placebo-controlled phase III study in Asian patients. *Journal of Diabetes Investigation*, *10*(5), 1299–1306.<https://doi.org/10.1111/jdi.13013>
14. Baba, M., Matsui, N., Kuroha, M., Wasaki, Y., & Ohwada, S. (2020). Long-term safety and efficacy of mirogabalin in Asian patients with diabetic peripheral neuropathic pain. *Journal of Diabetes Investigation*, *11*(3), 693–698.<https://doi.org/10.1111/jdi.13178>
15. Banerjee, S., & Argáez, C. (2019). *Multidisciplinary Treatment Programs for Patients with Acute or Subacute Pain: A Review of Clinical Effectiveness, Cost-Effectiveness, and Guidelines*. Canadian Agency for Drugs and Technologies in Health.<http://www.ncbi.nlm.nih.gov/books/NBK546002/>
16. Barohn, R. J., Gajewski, B., Pasnoor, M., Brown, A., Herbelin, L. L., Kimminau, K. S., Mudaranthakam, D. P., Jawdat, O., Dimachkie, M. M., Patient Assisted Intervention for Neuropathy: Comparison of Treatment in Real Life Situations (PAIN-CONTRoLS) Study Team, Iyadurai, S., Stino, A., Kissel, J., Pascuzzi, R., Brannagan, T., Wicklund, M., Ahmed, A., Walk, D., Smith, G., … Cash, T. (2021). Patient Assisted Intervention for Neuropathy: Comparison of Treatment in Real Life Situations (PAIN-CONTRoLS): Bayesian Adaptive Comparative Effectiveness Randomized Trial. *JAMA Neurology*, *78*(1), 68–76.<https://doi.org/10.1001/jamaneurol.2020.2590>
17. Baron, R., Binder, A., Attal, N., Casale, R., Dickenson, A. H., & Treede, R.-D. (2016). Neuropathic low back pain in clinical practice. *European Journal of Pain*, *20*(6), 861–873.<https://doi.org/10.1002/ejp.838>
18. Baron, Ralf, Likar, R., Martin-Mola, E., Blanco, F. J., Kennes, L., Müller, M., Falke, D., & Steigerwald, I. (2016). Effectiveness of Tapentadol Prolonged Release (PR) Compared with Oxycodone/Naloxone PR for the Management of Severe Chronic Low Back Pain with a Neuropathic Component: A Randomized, Controlled, Open-Label, Phase 3b/4 Study. *Pain Practice: The Official Journal of World Institute of Pain*, *16*(5), 580–599.<https://doi.org/10.1111/papr.12308>
19. Baron, R., Maier, C., Attal, N., Binder, A., Bouhassira, D., Cruccu, G., Finnerup, N. B., Haanpää, M., Hansson, P., Hüllemann, P., Jensen, T. S., Freynhagen, R., Kennedy, J. D., Magerl, W., Mainka, T., Reimer, M., Rice, A. S. C., Segerdahl, M., Serra, J., … on behalf of the German Neuropathic Pain Research Network (DFNS), and the E. (2017). Peripheral neuropathic pain: A mechanism-related organizing principle based on sensory profiles. *PAIN*, *158*(2), 261.<https://doi.org/10.1097/j.pain.0000000000000753>
20. Bauer, A., Hagenburger, J., Plank, T., Busch, V., & Greenlee, M. W. (2018). Mechanical Pain Thresholds and the Rubber Hand Illusion. *Frontiers in Psychology*, *9*, 712.<https://doi.org/10.3389/fpsyg.2018.00712>
21. Best, A. D., De Silva, R. K., Thomson, W. M., Tong, D. C., Cameron, C. M., & De Silva, H. L. (2017). Efficacy of Codeine When Added to Paracetamol (Acetaminophen) and Ibuprofen for Relief of Postoperative Pain After Surgical Removal of Impacted Third Molars: A Double-Blinded Randomized Control Trial. *Journal of Oral and Maxillofacial Surgery: Official Journal of the American Association of Oral and Maxillofacial Surgeons*, *75*(10), 2063–2069.<https://doi.org/10.1016/j.joms.2017.04.045>
22. Bonifácio de Assis, E. D., Martins, W. K. N., de Carvalho, C. D., Ferreira, C. M., Gomes, R., de Almeida Rodrigues, E. T., Meira, U. M., de Holanda, L. J., Lindquist, A. R., Morya, E., Mendes, C. K. T. T., de Assis, T. C. G., de Oliveira, E. A., & Andrade, S. M. (2022). Effects of rTMS and tDCS on neuropathic pain after brachial plexus injury: A randomized placebo-controlled pilot study. *Scientific Reports*, *12*(1), 1440.<https://doi.org/10.1038/s41598-022-05254-3>
23. Bradford, D., Stirling, A., Ernault, E., Liosatos, M., Tracy, K., Moseley, J., Blahunka, P., & Smith, M. D. (n.d.). *The MOBILE Study—A Phase IIa Enriched Enrollment Randomized Withdrawal Trial to Assess the Analgesic Efficacy and Safety of ASP8477, a Fatty Acid Amide Hydrolase Inhibitor, in Patients with Peripheral Neuropathic Pain*. Retrieved February 3, 2026, from<https://dx.doi.org/10.1093/pm/pnx046>
24. Celik, E. C., Erhan, B., Gunduz, B., & Lakse, E. (2013). The effect of low-frequency TENS in the treatment of neuropathic pain in patients with spinal cord injury. *Spinal Cord*, *51*(4), 334–337.<https://doi.org/10.1038/sc.2012.159>
25. Cervigni, M., Nasta, L., Schievano, C., Lampropoulou, N., & Ostardo, E. (2019). Micronized Palmitoylethanolamide-Polydatin Reduces the Painful Symptomatology in Patients with Interstitial Cystitis/Bladder Pain Syndrome. *BioMed Research International*, *2019*, 9828397.<https://doi.org/10.1155/2019/9828397>
26. Chao, M. T., Hurstak, E., Leonoudakis-Watts, K., Sidders, F., Pace, J., Hammer, H., & Wismer, B. (2019). Patient-Reported Outcomes of an Integrative Pain Management Program Implemented in a Primary Care Safety Net Clinic: A Quasi-experimental Study. *Journal of General Internal Medicine*, *34*(7), 1105–1107.<https://doi.org/10.1007/s11606-019-04868-0>
27. Chatterjee, P., Srivastava, A. K., Kumar, D. A., Chakrawarty, A., Khan, M. A., Ambashtha, A. K., Kumar, V., De Taboada, L., & Dey, A. B. (2019). Effect of deep tissue laser therapy treatment on peripheral neuropathic pain in older adults with type 2 diabetes: A pilot randomized clinical trial. *BMC Geriatrics*, *19*(1), 218.<https://doi.org/10.1186/s12877-019-1237-5>
28. Cox, E. R., Gajanand, T., Burton, N. W., Coombes, J. S., & Coombes, B. K. (2020). Effect of different exercise training intensities on musculoskeletal and neuropathic pain in inactive individuals with type 2 diabetes – Preliminary randomised controlled trial. *Diabetes Research and Clinical Practice*, *164*, 108168.<https://doi.org/10.1016/j.diabres.2020.108168>
29. Crasto, W., Altaf, Q.-A., Selvaraj, D. R., Jack, B., Patel, V., Nawaz, S., Murthy, N., Sukumar, N., Saravanan, P., & Tahrani, A. A. (2022). Frequency Rhythmic Electrical Modulation System (FREMS) to alleviate painful diabetic peripheral neuropathy: A pilot, randomised controlled trial (The FREMSTOP study). *Diabetic Medicine: A Journal of the British Diabetic Association*, *39*(3), e14710.<https://doi.org/10.1111/dme.14710>
30. Czarnetzki, C., Desmeules, J., Tessitore, E., Faundez, A., Chabert, J., Daali, Y., Fournier, R., Dupuis-Lozeron, E., Cedraschi, C., & Richard Tramèr, M. (2020). Perioperative intravenous low-dose ketamine for neuropathic pain after major lower back surgery: A randomized, placebo-controlled study. *European Journal of Pain*, *24*(3), 555–567.<https://doi.org/10.1002/ejp.1507>
31. da Silva Leal, M. V., Lima, M. O., Nicolau, R. A., de Carvallho, T. M. T., Abreu, J. A. de C., Pessoa, D. R., & Arisawa, E. A. L. S. (2020). Effect of Modified Laser Transcutaneous Irradiation on Pain and Quality of Life in Patients with Diabetic Neuropathy. *Photobiomodulation, Photomedicine, and Laser Surgery*, *38*(3), 138–144.<https://doi.org/10.1089/photob.2019.4714>
32. de Vos, C. C., Meier, K., Zaalberg, P. B., Nijhuis, H. J. A., Duyvendak, W., Vesper, J., Enggaard, T. P., & Lenders, M. W. P. M. (2014). Spinal cord stimulation in patients with painful diabetic neuropathy: A multicentre randomized clinical trial. *Pain*, *155*(11), 2426–2431.<https://doi.org/10.1016/j.pain.2014.08.031>
33. Demant, D. T., Lund, K., Finnerup, N. B., Vollert, J., Maier, C., Segerdahl, M. S., Jensen, T. S., & Sindrup, S. H. (2015). Pain relief with lidocaine 5% patch in localized peripheral neuropathic pain in relation to pain phenotype: A randomised, double-blind, and placebo-controlled, phenotype panel study. *Pain*, *156*(11), 2234–2244.<https://doi.org/10.1097/j.pain.0000000000000266>
34. Demant, D. T., Lund, K., Vollert, J., Maier, C., Segerdahl, M., Finnerup, N. B., Jensen, T. S., & Sindrup, S. H. (2014). The effect of oxcarbazepine in peripheral neuropathic pain depends on pain phenotype: A randomised, double-blind, placebo-controlled phenotype-stratified study. *Pain*, *155*(11), 2263–2273.<https://doi.org/10.1016/j.pain.2014.08.014>
35. Dhawan, S., Andrews, R., Kumar, L., Wadhwa, S., & Shukla, G. (2020). A Randomized Controlled Trial to Assess the Effectiveness of Muscle Strengthening and Balancing Exercises on Chemotherapy-Induced Peripheral Neuropathic Pain and Quality of Life Among Cancer Patients. *Cancer Nursing*, *43*(4), 269–280.<https://doi.org/10.1097/NCC.0000000000000693>
36. Dinat, N., Marinda, E., Moch, S., Rice, A. S. C., & Kamerman, P. R. (2015). Randomized, Double-Blind, Crossover Trial of Amitriptyline for Analgesia in Painful HIV-Associated Sensory Neuropathy. *PloS One*, *10*(5), e0126297.<https://doi.org/10.1371/journal.pone.0126297>
37. Dou, L., Lin, H., Wang, K., Zhu, G., Zou, X., Chang, E., & Zhu, Y. (2017). Long non-coding RNA CCAT1 modulates neuropathic pain progression through sponging miR-155. *Oncotarget*, *8*(52), 89949–89957.<https://doi.org/10.18632/oncotarget.21192>
38. Eibach, L., Scheffel, S., Cardebring, M., Lettau, M., Özgür Celik, M., Morguet, A., Roehle, R., & Stein, C. (2021). Cannabidivarin for HIV-Associated Neuropathic Pain: A Randomized, Blinded, Controlled Clinical Trial. *Clinical Pharmacology and Therapeutics*, *109*(4), 1055–1062.<https://doi.org/10.1002/cpt.2016>
39. Eisenberg, E., Burstein, Y., Suzan, E., Treister, R., & Aviram, J. (2015). Spinal cord stimulation attenuates temporal summation in patients with neuropathic pain. *Pain*, *156*(3), 381–385.<https://doi.org/10.1097/01.j.pain.0000460342.69718.a2>
40. Estores, I., Chen, K., Jackson, B., Lao, L., & Gorman, P. H. (2017). Auricular acupuncture for spinal cord injury related neuropathic pain: A pilot controlled clinical trial. *The Journal of Spinal Cord Medicine*, *40*(4), 432–438.<https://doi.org/10.1080/10790268.2016.1141489>
41. Etienne, R., Laurent, M., Henry, A., Bioy, A., Salleron, J., Schohn, C. H., & Cretineau, N. (2021). Interest of a standardized hypnotic message for the reduction of pain and anxiety in cancer patients treated by capsaicin patch for neuropathic pain: A randomized controlled trial. *BMC Complementary Medicine and Therapies*, *21*(1), 154.<https://doi.org/10.1186/s12906-021-03329-8>
42. Evangelista, M. C., Monteiro, B. P., & Steagall, P. V. (2022). Measurement properties of grimace scales for pain assessment in nonhuman mammals: A systematic review. *Pain*, *163*(6), e697–e714.<https://doi.org/10.1097/j.pain.0000000000002474>
43. Fallon, M., Giusti, R., Aielli, F., Hoskin, P., Rolke, R., Sharma, M., Ripamonti, C. I., & ESMO Guidelines Committee. (2018). Management of cancer pain in adult patients: ESMO Clinical Practice Guidelines. *Annals of Oncology: Official Journal of the European Society for Medical Oncology*, *29*(Suppl 4), iv166–iv191.<https://doi.org/10.1093/annonc/mdy152>
44. Foley, P., Parker, R. A., de Angelis, F., Connick, P., Chandran, S., Young, C., Weir, C. J., Chataway, J., & MS-SMART Investigators. (2022). Efficacy of Fluoxetine, Riluzole and Amiloride in treating neuropathic pain associated with secondary progressive multiple sclerosis. Pre-specified analysis of the MS-SMART double-blind randomised placebo-controlled trial. *Multiple Sclerosis and Related Disorders*, *63*, 103925.<https://doi.org/10.1016/j.msard.2022.103925>
45. Galhardoni, R., Aparecida da Silva, V., García-Larrea, L., Dale, C., Baptista, A. F., Barbosa, L. M., Menezes, L. M. B., de Siqueira, S. R. D. T., Valério, F., Rosi, J., de Lima Rodrigues, A. L., Reis Mendes Fernandes, D. T., Lorencini Selingardi, P. M., Marcolin, M. A., Duran, F. L. de S., Ono, C. R., Lucato, L. T., Fernandes, A. M. B. L., da Silva, F. E. F., … Ciampi de Andrade, D. (2019). Insular and anterior cingulate cortex deep stimulation for central neuropathic pain: Disassembling the percept of pain. *Neurology*, *92*(18), e2165–e2175.<https://doi.org/10.1212/WNL.0000000000007396>
46. Ganesan, P., Manjini, K. J., & Bathala Vedagiri, S. C. (2022). Effect of Music on Pain, Anxiety and Physiological Parameters among Postoperative Sternotomy Patients: A Randomized Controlled Trial. *Journal of Caring Sciences*, *11*(3), 139–147.<https://doi.org/10.34172/jcs.2022.18>
47. Gao, Y., Guo, X., Han, P., Li, Q., Yang, G., Qu, S., Yue, L., Wang, C.-N., Skljarevski, V., Dueñas, H., Raskin, J., & Gu, L. (2015). Treatment of patients with diabetic peripheral neuropathic pain in China: A double-blind randomised trial of duloxetine vs. placebo. *International Journal of Clinical Practice*, *69*(9), 957–966.<https://doi.org/10.1111/ijcp.12641>
48. Garrow, A. P., Xing, M., Vere, J., Verrall, B., Wang, L., & Jude, E. B. (2014). Role of acupuncture in the management of diabetic painful neuropathy (DPN): A pilot RCT. *Acupuncture in Medicine: Journal of the British Medical Acupuncture Society*, *32*(3), 242–249.<https://doi.org/10.1136/acupmed-2013-010495>
49. Geerts, M., de Greef, B. T. A., Sopacua, M., van Kuijk, S. M. J., Hoeijmakers, J. G. J., Faber, C. G., & Merkies, I. S. J. (2021). Intravenous Immunoglobulin Therapy in Patients With Painful Idiopathic Small Fiber Neuropathy. *Neurology*, *96*(20), e2534–e2545.<https://doi.org/10.1212/WNL.0000000000011919>
50. Geraets, C. N. W., van Beilen, M., van Dijk, M., Kleijer, H., Köhne, C., van der Hoeven, J. H., Groen, G. J., Ćurčić-Blake, B., Schoevers, R. A., Maurits, N. M., & Kortekaas, R. (2019). Lack of analgesic effects of transcranial pulsed electromagnetic field stimulation in neuropathic pain patients: A randomized double-blind crossover trial. *Neuroscience Letters*, *699*, 212–216.<https://doi.org/10.1016/j.neulet.2019.01.051>
51. Gilmore, C., Ilfeld, B., Rosenow, J., Li, S., Desai, M., Hunter, C., Rauck, R., Kapural, L., Nader, A., Mak, J., Cohen, S., Crosby, N., & Boggs, J. (2019). Percutaneous peripheral nerve stimulation for the treatment of chronic neuropathic postamputation pain: A multicenter, randomized, placebo-controlled trial. *Regional Anesthesia and Pain Medicine*, *44*(6), 637–645.<https://doi.org/10.1136/rapm-2018-100109>
52. Gilron, I., Tu, D., Holden, R. R., Jackson, A. C., & DuMerton-Shore, D. (2015). Combination of morphine with nortriptyline for neuropathic pain. *Pain*, *156*(8), 1440–1448.<https://doi.org/10.1097/j.pain.0000000000000149>
53. Gok Metin, Z., Arikan Donmez, A., Izgu, N., Ozdemir, L., & Arslan, I. E. (2017). Aromatherapy Massage for Neuropathic Pain and Quality of Life in Diabetic Patients. *Journal of Nursing Scholarship: An Official Publication of Sigma Theta Tau International Honor Society of Nursing*, *49*(4), 379–388.<https://doi.org/10.1111/jnu.12300>
54. Goldlust, S. A., Kavoosi, Mojgan, Nezzer, J., Kavoosi, Mehran, Korz, W., & Deck, K. (2021). Tetrodotoxin for Chemotherapy-Induced Neuropathic Pain: A Randomized, Double-Blind, Placebo-Controlled, Parallel-Dose Finding Trial. *Toxins*, *13*(4), 235.<https://doi.org/10.3390/toxins13040235>
55. González-Duarte, A., Lem, M., Díaz-Díaz, E., Castillo, C., & Cárdenas-Soto, K. (2016). The Efficacy of Pregabalin in the Treatment of Prediabetic Neuropathic Pain. *The Clinical Journal of Pain*, *32*(11), 927–932.<https://doi.org/10.1097/AJP.0000000000000339>
56. Gül, Ş. K., Tepetam, H., & Gül, H. L. (2020). Duloxetine and pregabalin in neuropathic pain of lung cancer patients. *Brain and Behavior*, *10*(3), e01527.<https://doi.org/10.1002/brb3.1527>
57. Haanpää, M., Cruccu, G., Nurmikko, T. J., McBride, W. T., Docu Axelarad, A., Bosilkov, A., Chambers, C., Ernault, E., & Abdulahad, A. K. (2016). Capsaicin 8% patch versus oral pregabalin in patients with peripheral neuropathic pain. *European Journal of Pain*, *20*(2), 316–328.<https://doi.org/10.1002/ejp.731>
58. Hagenacker, T., Bude, V., Naegel, S., Holle, D., Katsarava, Z., Diener, H.-C., & Obermann, M. (2014). Patient-conducted anodal transcranial direct current stimulation of the motor cortex alleviates pain in trigeminal neuralgia. *The Journal of Headache and Pain*, *15*(1), 78.<https://doi.org/10.1186/1129-2377-15-78>
59. Hamani, C., Fonoff, E. T., Parravano, D. C., Silva, V. A., Galhardoni, R., Monaco, B. A., Navarro, J., Yeng, L. T., Teixeira, M. J., & de Andrade, D. C. (2021). Motor cortex stimulation for chronic neuropathic pain: Results of a double-blind randomized study. *Brain: A Journal of Neurology*, *144*(10), 2994–3004.<https://doi.org/10.1093/brain/awab189>
60. Han, Z.-A., Song, D. H., Oh, H.-M., & Chung, M. E. (2016). Botulinum toxin type A for neuropathic pain in patients with spinal cord injury. *Annals of Neurology*, *79*(4), 569–578.<https://doi.org/10.1002/ana.24605>
61. Hassanien, M., Elawamy, A., Kamel, E. Z., Khalifa, W. A., Abolfadl, G. M., Roushdy, A. S. I., El Zohne, R. A., & Makarem, Y. S. (2020). Perineural Platelet-Rich Plasma for Diabetic Neuropathic Pain, Could It Make a Difference? *Pain Medicine*, *21*(4), 757–765.<https://doi.org/10.1093/pm/pnz140>
62. Haumann, J., Geurts, J. W., van Kuijk, S. M. J., Kremer, B., Joosten, E. A., & van den Beuken-van Everdingen, M. H. J. (2016). Methadone is superior to fentanyl in treating neuropathic pain in patients with head-and-neck cancer. *European Journal of Cancer*, *65*, 121–129.<https://doi.org/10.1016/j.ejca.2016.06.025>
63. Hearn, J. H., Cotter, I., & Finlay, K. A. (2019). Efficacy of Internet-Delivered Mindfulness for Improving Depression in Caregivers of People With Spinal Cord Injuries and Chronic Neuropathic Pain: A Randomized Controlled Feasibility Trial. *Archives of Physical Medicine and Rehabilitation*, *100*(1), 17–25.<https://doi.org/10.1016/j.apmr.2018.08.182>
64. Herring, W. J., Roth, T., Krystal, A. D., & Michelson, D. (2019). Orexin receptor antagonists for the treatment of insomnia and potential treatment of other neuropsychiatric indications. *Journal of Sleep Research*, *28*(2), e12782.<https://doi.org/10.1111/jsr.12782>
65. Heutink, M., Post, M. W., Luthart, P., Schuitemaker, M., Slangen, S., Sweers, J., Vlemmix, L., & Lindeman, E. (2014). Long-term outcomes of a multidisciplinary cognitive behavioural programme for coping with chronic neuropathic spinal cord injury pain. *Journal of Rehabilitation Medicine*, *46*(6), 540–545.<https://doi.org/10.2340/16501977-1798>
66. Heydari, M., Homayouni, K., Hashempur, M. H., & Shams, M. (2016). Topical Citrullus colocynthis (bitter apple) extract oil in painful diabetic neuropathy: A double-blind randomized placebo-controlled clinical trial. *Journal of Diabetes*, *8*(2), 246–252.<https://doi.org/10.1111/1753-0407.12287>
67. Hincker, A., Frey, K., Rao, L., Wagner-Johnston, N., Ben Abdallah, A., Tan, B., Amin, M., Wildes, T., Shah, R., Karlsson, P., Bakos, K., Kosicka, K., Kagan, L., & Haroutounian, S. (2019). Somatosensory predictors of response to pregabalin in painful chemotherapy-induced peripheral neuropathy: A randomized, placebo-controlled, crossover study. *Pain*, *160*(8), 1835–1846.<https://doi.org/10.1097/j.pain.0000000000001577>
68. Hoggart, B., Ratcliffe, S., Ehler, E., Simpson, K. H., Hovorka, J., Lejčko, J., Taylor, L., Lauder, H., & Serpell, M. (2015). A multicentre, open-label, follow-on study to assess the long-term maintenance of effect, tolerance and safety of THC/CBD oromucosal spray in the management of neuropathic pain. *Journal of Neurology*, *262*(1), 27–40.<https://doi.org/10.1007/s00415-014-7502-9>
69. Hong, L., Zhang, J., & Shen, J. (2015). Clinical efficacy of different doses of lipo-prostaglandin E1 in the treatment of painful diabetic peripheral neuropathy. *Journal of Diabetes and Its Complications*, *29*(8), 1283–1286.<https://doi.org/10.1016/j.jdiacomp.2015.08.001>
70. Hosomi, K., Shimokawa, T., Ikoma, K., Nakamura, Y., Sugiyama, K., Ugawa, Y., Uozumi, T., Yamamoto, T., & Saitoh, Y. (2013). Daily repetitive transcranial magnetic stimulation of primary motor cortex for neuropathic pain: A randomized, multicenter, double-blind, crossover, sham-controlled trial. *Pain*, *154*(7), 1065–1072.<https://doi.org/10.1016/j.pain.2013.03.016>
71. Hosomi, K., Sugiyama, K., Nakamura, Y., Shimokawa, T., Oshino, S., Goto, Y., Mano, T., Shimizu, T., Yanagisawa, T., Saitoh, Y., & TEN-P11-01 investigators. (2020). A randomized controlled trial of 5 daily sessions and continuous trial of 4 weekly sessions of repetitive transcranial magnetic stimulation for neuropathic pain. *Pain*, *161*(2), 351–360.<https://doi.org/10.1097/j.pain.0000000000001712>
72. Huffman, C., Stacey, B. R., Tuchman, M., Burbridge, C., Li, C., Parsons, B., Pauer, L., Scavone, J. M., Behar, R., & Yurkewicz, L. (2015). Efficacy and Safety of Pregabalin in the Treatment of Patients With Painful Diabetic Peripheral Neuropathy and Pain on Walking. *The Clinical Journal of Pain*, *31*(11), 946–958.<https://doi.org/10.1097/AJP.0000000000000198>
73. Hussain, N., & Said, A. S. A. (2019). Mindfulness-Based Meditation Versus Progressive Relaxation Meditation: Impact on Chronic Pain in Older Female Patients With Diabetic Neuropathy. *Journal of Evidence-Based Integrative Medicine*, *24*, 2515690X19876599.<https://doi.org/10.1177/2515690X19876599>
74. Hutmacher, M. M., Frame, B., Miller, R., Truitt, K., & Merante, D. (2016). Exposure-response modeling of average daily pain score, and dizziness and somnolence, for mirogabalin (DS-5565) in patients with diabetic peripheral neuropathic pain. *Journal of Clinical Pharmacology*, *56*(1), 67–77.<https://doi.org/10.1002/jcph.567>
75. Hwang, C. J., Lee, J. H., Kim, J.-H., Min, S. H., Park, K.-W., Seo, H.-Y., & Song, K.-S. (2019). Gabapentin versus Transdermal Fentanyl Matrix for the Alleviation of Chronic Neuropathic Pain of Radicular Origin: A Randomized Blind Multicentered Parallel-Group Noninferiority Trial. *Pain Research & Management*, *2019*, 4905013.<https://doi.org/10.1155/2019/4905013>
76. Irving, G., Tanenberg, R. J., Raskin, J., Risser, R. C., & Malcolm, S. (2014). Comparative safety and tolerability of duloxetine vs. Pregabalin vs. Duloxetine plus gabapentin in patients with diabetic peripheral neuropathic pain. *International Journal of Clinical Practice*, *68*(9), 1130–1140.<https://doi.org/10.1111/ijcp.12452>
77. Ivanishvili, Z., Poologaindran, A., & Honey, C. R. (2017). Cyclization of Motor Cortex Stimulation for Neuropathic Pain: A Prospective, Randomized, Blinded Trial. *Neuromodulation: Journal of the International Neuromodulation Society*, *20*(5), 497–503.<https://doi.org/10.1111/ner.12610>
78. Izgu, N., Gok Metin, Z., Karadas, C., Ozdemir, L., Metinarikan, N., & Corapcıoglu, D. (2020). Progressive Muscle Relaxation and Mindfulness Meditation on Neuropathic Pain, Fatigue, and Quality of Life in Patients With Type 2 Diabetes: A Randomized Clinical Trial. *Journal of Nursing Scholarship: An Official Publication of Sigma Theta Tau International Honor Society of Nursing*, *52*(5), 476–487.<https://doi.org/10.1111/jnu.12580>
79. Jensen, T. S., Høye, K., Fricová, J., Vanelderen, P., Ernault, E., Siciliano, T., & Marques, S. (2014). Tolerability of the capsaicin 8% patch following pretreatment with lidocaine or tramadol in patients with peripheral neuropathic pain: A multicentre, randomized, assessor-blinded study. *European Journal of Pain*, *18*(9), 1240–1247.<https://doi.org/10.1002/j.1532-2149.2014.00479.x>
80. Jiang, J., Li, Y., Shen, Q., Rong, X., Huang, X., Li, H., Zhou, L., Mai, H.-Q., Zheng, D., Chen, M.-Y., Xu, Y., Li, J., Hui, X., Simone, C. B., Gaertner, J., Argyriou, A. A., Chow, E., Chen, P., & Tang, Y. (2019). Effect of Pregabalin on Radiotherapy-Related Neuropathic Pain in Patients With Head and Neck Cancer: A Randomized Controlled Trial. *Journal of Clinical Oncology: Official Journal of the American Society of Clinical Oncology*, *37*(2), 135–143.<https://doi.org/10.1200/JCO.18.00896>
81. Jordan, M., & Richardson, E. J. (2016). Effects of Virtual Walking Treatment on Spinal Cord Injury-Related Neuropathic Pain: Pilot Results and Trends Related to Location of Pain and at-level Neuronal Hypersensitivity. *American Journal of Physical Medicine & Rehabilitation*, *95*(5), 390–396.<https://doi.org/10.1097/PHM.0000000000000417>
82. Jung, J.-M., Chung, C. K., Kim, C. H., Yang, S. H., & Choi, Y. (2020). Comparison of the use of opioids only and pregabalin add-on for the treatment of neuropathic pain in cervical myelopathy patients: A pilot trial. *Scientific Reports*, *10*(1), 8120.<https://doi.org/10.1038/s41598-020-65108-8>
83. Karmakar, S., Rashidian, H., Chan, C., Liu, C., & Toth, C. (2014). Investigating the role of neuropathic pain relief in decreasing gait variability in diabetes mellitus patients with neuropathic pain: A randomized, double-blind crossover trial. *Journal of Neuroengineering and Rehabilitation*, *11*, 125.<https://doi.org/10.1186/1743-0003-11-125>
84. Kato, J., Baba, M., Kuroha, M., Kakehi, Y., Murayama, E., Wasaki, Y., & Ohwada, S. (2021). Safety and Efficacy of Mirogabalin for Peripheral Neuropathic Pain: Pooled Analysis of Two Pivotal Phase III Studies. *Clinical Therapeutics*, *43*(5), 822-835.e16.<https://doi.org/10.1016/j.clinthera.2021.03.015>
85. Kaur, J., Ghosh, S., Sahani, A. K., & Sinha, J. K. (2020). Mental Imagery as a Rehabilitative Therapy for Neuropathic Pain in People With Spinal Cord Injury: A Randomized Controlled Trial. *Neurorehabilitation and Neural Repair*, *34*(11), 1038–1049.<https://doi.org/10.1177/1545968320962498>
86. Keene, D. J., Knight, R., Bruce, J., Dutton, S. J., Tutton, E., Achten, J., & Costa, M. L. (2021). Chronic pain with neuropathic characteristics after surgery for major trauma to the lower limb: Prevalence, predictors, and association with pain severity, disability, and quality of life in the UK WHiST trial. *The Bone & Joint Journal*, *103-B*(6), 1047–1054.<https://doi.org/10.1302/0301-620X.103B.BJJ-2020-2204.R1>
87. Kerckhove, N., Pereira, B., Soriot-Thomas, S., Alchaar, H., Deleens, R., Hieng, V. S., Serra, E., Lanteri-Minet, M., Arcagni, P., Picard, P., Lefebvre-Kuntz, D., Maindet, C., Mick, G., Balp, L., Lucas, C., Creach, C., Letellier, M., Martinez, V., Navez, M., … Eschalier, A. (2018). Efficacy and safety of a T-type calcium channel blocker in patients with neuropathic pain: A proof-of-concept, randomized, double-blind and controlled trial. *European Journal of Pain*, *22*(7), 1321–1330.<https://doi.org/10.1002/ejp.1221>
88. Kerckhove, N., Scanzi, J., Pereira, B., Ardid, D., & Dapoigny, M. (2017). Assessment of the effectiveness and safety of ethosuximide in the treatment of abdominal pain related to irritable bowel syndrome – IBSET: Protocol of a randomised, parallel, controlled, double-blind and multicentre trial. *BMJ Open*, *7*(7), e015380.<https://doi.org/10.1136/bmjopen-2016-015380>
89. Kersten, C., Cameron, M. G., Bailey, A. G., Fallon, M. T., Laird, B. J., Paterson, V., Mitchell, R., Fleetwood-Walker, S. M., Daly, F., & Mjåland, S. (2019). Relief of Neuropathic Pain Through Epidermal Growth Factor Receptor Inhibition: A Randomized Proof-of-Concept Trial. *Pain Medicine*, *20*(12), 2495–2505.<https://doi.org/10.1093/pm/pnz101>
90. Khan, J. S., Hodgson, N., Choi, S., Reid, S., Paul, J. E., Hong, N. J. L., Holloway, C., Busse, J. W., Gilron, I., Buckley, D. N., McGillion, M., Clarke, H., Katz, J., Mackey, S., Avram, R., Pohl, K., Rao-Melacini, P., & Devereaux, P. J. (2019). Perioperative Pregabalin and Intraoperative Lidocaine Infusion to Reduce Persistent Neuropathic Pain After Breast Cancer Surgery: A Multicenter, Factorial, Randomized, Controlled Pilot Trial. *The Journal of Pain*, *20*(8), 980–993.<https://doi.org/10.1016/j.jpain.2019.02.010>
91. Khasbage, S., Shukla, R., Sharma, P., & Singh, S. (2021). A randomized control trial of duloxetine and gabapentin in painful diabetic neuropathy. *Journal of Diabetes*, *13*(7), 532–541.<https://doi.org/10.1111/1753-0407.13148>
92. Khedr, E. M., Kotb, H. I., Mostafa, M. G., Mohamad, M. F., Amr, S. A., Ahmed, M. A., Karim, A. A., & Kamal, S. M. M. (2015). Repetitive transcranial magnetic stimulation in neuropathic pain secondary to malignancy: A randomized clinical trial. *European Journal of Pain*, *19*(4), 519–527.<https://doi.org/10.1002/ejp.576>
93. Kim, Y. H., Lee, P. B., & Oh, T. K. (2015). Is magnesium sulfate effective for pain in chronic postherpetic neuralgia patients comparing with ketamine infusion therapy? *Journal of Clinical Anesthesia*, *27*(4), 296–300.<https://doi.org/10.1016/j.jclinane.2015.02.006>
94. Kim, Y.-C., Castañeda, A. M., Lee, C.-S., Jin, H.-S., Park, K. S., & Moon, J. Y. (2018). Efficacy and Safety of Lidocaine Infusion Treatment for Neuropathic Pain: A Randomized, Double-Blind, and Placebo-Controlled Study. *Regional Anesthesia and Pain Medicine*, *43*(4), 415–424.<https://doi.org/10.1097/AAP.0000000000000741>
95. Knoerl, R., Chornoby, Z., & Smith, E. M. L. (2018). Estimating the Frequency, Severity, and Clustering of SPADE Symptoms in Chronic Painful Chemotherapy-Induced Peripheral Neuropathy. *Pain Management Nursing: Official Journal of the American Society of Pain Management Nurses*, *19*(4), 354–365.<https://doi.org/10.1016/j.pmn.2018.01.001>
96. Kulkantrakorn, K., Chomjit, A., Sithinamsuwan, P., Tharavanij, T., Suwankanoknark, J., & Napunnaphat, P. (2019). 0.075% capsaicin lotion for the treatment of painful diabetic neuropathy: A randomized, double-blind, crossover, placebo-controlled trial. *Journal of Clinical Neuroscience: Official Journal of the Neurosurgical Society of Australasia*, *62*, 174–179.<https://doi.org/10.1016/j.jocn.2018.11.036>
97. Kulkantrakorn, K., Lorsuwansiri, C., & Meesawatsom, P. (2013). 0.025% capsaicin gel for the treatment of painful diabetic neuropathy: A randomized, double-blind, crossover, placebo-controlled trial. *Pain Practice: The Official Journal of World Institute of Pain*, *13*(6), 497–503.<https://doi.org/10.1111/papr.12013>
98. Kumru, H., Benito-Penalva, J., Kofler, M., & Vidal, J. (2018). Analgesic effect of intrathecal baclofen bolus on neuropathic pain in spinal cord injury patients. *Brain Research Bulletin*, *140*, 205–211.<https://doi.org/10.1016/j.brainresbull.2018.05.013>
99. Lagas, I. F., van der Vlist, A. C., van Oosterom, R. F., van Veldhoven, P. L. J., Waarsing, J. H., Bierma-Zeinstra, S. M. A., Verhaar, J. A. N., & de Vos, R.-J. (2021). Are pain coping strategies and neuropathic pain associated with a worse outcome after conservative treatment for Achilles tendinopathy? A prospective cohort study. *Journal of Science and Medicine in Sport*, *24*(9), 871–875.<https://doi.org/10.1016/j.jsams.2021.04.001>
100. Langford, R. M., Mares, J., Novotna, A., Vachova, M., Novakova, I., Notcutt, W., & Ratcliffe, S. (2013). A double-blind, randomized, placebo-controlled, parallel-group study of THC/CBD oromucosal spray in combination with the existing treatment regimen, in the relief of central neuropathic pain in patients with multiple sclerosis. *Journal of Neurology*, *260*(4), 984–997.<https://doi.org/10.1007/s00415-012-6739-4>
101. Lee, M.-K., Jeon, Y., Choi, S. S., Lee, P. B., Kim, Y.-C., Suh, J. H., Sim, S. E., Song, S. O., Ko, Y., Yu, J. M., Min, K., & Lee, J.-H. (2020). Efficacy and Safety of the Controlled-release Pregabalin Tablet (GLA5PR GLARS-NF1) and Immediate-release Pregabalin Capsule for Peripheral Neuropathic Pain: A Multicenter, Randomized, Double-blind, Parallel-group, Active-controlled, Phase III Clinical Trial. *Clinical Therapeutics*, *42*(12), 2266–2279.<https://doi.org/10.1016/j.clinthera.2020.10.009>
102. Lee, S., Kim, J.-H., Shin, K.-M., Kim, J.-E., Kim, T.-H., Kang, K.-W., Lee, M., Jung, S.-Y., Shin, M.-S., Kim, A.-R., Park, H.-J., Hong, K.-E., & Choi, S.-M. (2013). Electroacupuncture to treat painful diabetic neuropathy: Study protocol for a three-armed, randomized, controlled pilot trial. *Trials*, *14*, 225.<https://doi.org/10.1186/1745-6215-14-225>
103. Lewis, G. N., Rice, D. A., Kluger, M., & McNair, P. J. (2018). Transcranial direct current stimulation for upper limb neuropathic pain: A double-blind randomized controlled trial. *European Journal of Pain*, *22*(7), 1312–1320.<https://doi.org/10.1002/ejp.1220>
104. Liao, C.-D., Rau, C.-L., Liou, T.-H., Tsauo, J.-Y., & Lin, L.-F. (2017). Effects of Linearly Polarized Near-Infrared Irradiation Near the Stellate Ganglion Region on Pain and Heart Rate Variability in Patients with Neuropathic Pain. *Pain Medicine*, *18*(3), 488–503.<https://doi.org/10.1093/pm/pnw145>
105. Lindholm, P., Lamusuo, S., Taiminen, T., Pesonen, U., Lahti, A., Virtanen, A., Forssell, H., Hietala, J., Hagelberg, N., Pertovaara, A., Parkkola, R., & Jääskeläinen, S. (2015). Right secondary somatosensory cortex-a promising novel target for the treatment of drug-resistant neuropathic orofacial pain with repetitive transcranial magnetic stimulation. *Pain*, *156*(7), 1276–1283.<https://doi.org/10.1097/j.pain.0000000000000175>
106. Lipone, P., Ehler, E., Nastaj, M., Palka-Kisielowska, I., Cruccu, G., Truini, A., Di Loreto, G., Del Vecchio, A., Pochiero, I., Comandini, A., Calisti, F., & Cattaneo, A. (2020). Efficacy and Safety of Low Doses of Trazodone in Patients Affected by Painful Diabetic Neuropathy and Treated with Gabapentin: A Randomized Controlled Pilot Study. *CNS Drugs*, *34*(11), 1177–1189.<https://doi.org/10.1007/s40263-020-00760-2>
107. Liu, W.-Q., Kanungo, A., & Toth, C. (2014). Equivalency of tricyclic antidepressants in open-label neuropathic pain study. *Acta Neurologica Scandinavica*, *129*(2), 132–141.<https://doi.org/10.1111/ane.12169>
108. Lynch, M. E., Cesar-Rittenberg, P., & Hohmann, A. G. (2014). A double-blind, placebo-controlled, crossover pilot trial with extension using an oral mucosal cannabinoid extract for treatment of chemotherapy-induced neuropathic pain. *Journal of Pain and Symptom Management*, *47*(1), 166–173.<https://doi.org/10.1016/j.jpainsymman.2013.02.018>
109. Ma, S.-M., Ni, J.-X., Li, X.-Y., Yang, L.-Q., Guo, Y.-N., & Tang, Y.-Z. (2015). High-Frequency Repetitive Transcranial Magnetic Stimulation Reduces Pain in Postherpetic Neuralgia. *Pain Medicine*, *16*(11), 2162–2170.<https://doi.org/10.1111/pme.12832>
110. Maharaj, S. S., & Yakasai, A. M. (2018). Does a Rehabilitation Program of Aerobic and Progressive Resisted Exercises Influence HIV-Induced Distal Neuropathic Pain? *American Journal of Physical Medicine & Rehabilitation*, *97*(5), 364–369.<https://doi.org/10.1097/PHM.0000000000000866>
111. Maihöfner, C., & Heskamp, M.-L. (2014). Treatment of peripheral neuropathic pain by topical capsaicin: Impact of pre-existing pain in the QUEPP-study. *European Journal of Pain (London, England)*, *18*(5), 671–679.<https://doi.org/10.1002/j.1532-2149.2013.00415.x>
112. Markman, J., Resnick, M., Greenberg, S., Katz, N., Yang, R., Scavone, J., Whalen, E., Gregorian, G., Parsons, B., & Knapp, L. (2018). Efficacy of pregabalin in post-traumatic peripheral neuropathic pain: A randomized, double-blind, placebo-controlled phase 3 trial. *Journal of Neurology*, *265*(12), 2815–2824.<https://doi.org/10.1007/s00415-018-9063-9>
113. Matsuoka, H., Ishiki, H., Iwase, S., Koyama, A., Kawaguchi, T., Kizawa, Y., Morita, T., Matsuda, Y., Miyaji, T., Ariyoshi, K., & Yamaguchi, T. (2017). Study protocol for a multi-institutional, randomised, double-blinded, placebo-controlled phase III trial investigating additive efficacy of duloxetine for neuropathic cancer pain refractory to opioids and gabapentinoids: The DIRECT study. *BMJ Open*, *7*(8), e017280.<https://doi.org/10.1136/bmjopen-2017-017280>
114. Matsuoka, H., Iwase, S., Miyaji, T., Kawaguchi, T., Ariyoshi, K., Oyamada, S., Satomi, E., Ishiki, H., Hasuo, H., Sakuma, H., Tokoro, A., Matsuda, Y., Tahara, K., Otani, H., Ohtake, Y., Tsukuura, H., Matsumoto, Y., Hasegawa, Y., Kataoka, Y., … Koyama, A. (2020). Predictors of duloxetine response in patients with neuropathic cancer pain: A secondary analysis of a randomized controlled trial-JORTC-PAL08 (DIRECT) study. *Supportive Care in Cancer: Official Journal of the Multinational Association of Supportive Care in Cancer*, *28*(6), 2931–2939.<https://doi.org/10.1007/s00520-019-05138-9>
115. Matsuoka, H., Iwase, S., Miyaji, T., Kawaguchi, T., Ariyoshi, K., Oyamada, S., Satomi, E., Ishiki, H., Hasuo, H., Sakuma, H., Tokoro, A., Shinomiya, T., Otani, H., Ohtake, Y., Tsukuura, H., Matsumoto, Y., Hasegawa, Y., Kataoka, Y., Otsuka, M., … Yamaguchi, T. (2019). Additive Duloxetine for Cancer-Related Neuropathic Pain Nonresponsive or Intolerant to Opioid-Pregabalin Therapy: A Randomized Controlled Trial (JORTC-PAL08). *Journal of Pain and Symptom Management*, *58*(4), 645–653.<https://doi.org/10.1016/j.jpainsymman.2019.06.020>
116. McDonnell, A., Collins, S., Ali, Z., Iavarone, L., Surujbally, R., Kirby, S., & Butt, R. P. (2018). Efficacy of the Nav1.7 blocker PF-05089771 in a randomised, placebo-controlled, double-blind clinical study in subjects with painful diabetic peripheral neuropathy. *Pain*, *159*(8), 1465–1476.<https://doi.org/10.1097/j.pain.0000000000001227>
117. Mehta, N., Bucior, I., Bujanover, S., Shah, R., & Gulati, A. (2016). Relationship between pain relief, reduction in pain-associated sleep interference, and overall impression of improvement in patients with postherpetic neuralgia treated with extended-release gabapentin. *Health and Quality of Life Outcomes*, *14*, 54.<https://doi.org/10.1186/s12955-016-0456-0>
118. Mekhail, N. A., Argoff, C. E., Taylor, R. S., Nasr, C., Caraway, D. L., Gliner, B. E., Subbaroyan, J., & Brooks, E. S. (2020). High-frequency spinal cord stimulation at 10 kHz for the treatment of painful diabetic neuropathy: Design of a multicenter, randomized controlled trial (SENZA-PDN). *Trials*, *21*, 87.<https://doi.org/10.1186/s13063-019-4007-y>
119. Merante, D., Rosenstock, J., Sharma, U., Feins, K., Hsu, C., Vinik, A., & DS-5565-A-U201 US Phase 2 Study Investigators. (2017). Efficacy of Mirogabalin (DS-5565) on Patient-Reported Pain and Sleep Interference in Patients with Diabetic Neuropathic Pain: Secondary Outcomes of a Phase II Proof-of-Concept Study. *Pain Medicine*, *18*(11), 2198–2207.<https://doi.org/10.1093/pm/pnw342>
120. Mimenza Alvarado, A., & Aguilar Navarro, S. (2016). Clinical Trial Assessing the Efficacy of Gabapentin Plus B Complex (B1/B12) versus Pregabalin for Treating Painful Diabetic Neuropathy. *Journal of Diabetes Research*, *2016*, 4078695.<https://doi.org/10.1155/2016/4078695>
121. Min, K., Oh, Y., Lee, S.-H., & Ryu, J. S. (2016). Symptom-Based Treatment of Neuropathic Pain in Spinal Cord-Injured Patients: A Randomized Crossover Clinical Trial. *American Journal of Physical Medicine & Rehabilitation*, *95*(5), 330–338.<https://doi.org/10.1097/PHM.0000000000000382>
122. Mori, N., Hosomi, K., Nishi, A., Oshino, S., Kishima, H., & Saitoh, Y. (2022). Analgesic Effects of Repetitive Transcranial Magnetic Stimulation at Different Stimulus Parameters for Neuropathic Pain: A Randomized Study. *Neuromodulation: Journal of the International Neuromodulation Society*, *25*(4), 520–527.<https://doi.org/10.1111/ner.13328>
123. Motilal, S., & Maharaj, R. G. (2013). Nutmeg extracts for painful diabetic neuropathy: A randomized, double-blind, controlled study. *Journal of Alternative and Complementary Medicine*, *19*(4), 347–352.<https://doi.org/10.1089/acm.2012.0016>
124. Moulin, D. E., Morley-Forster, P. K., Pirani, Z., Rohfritsch, C., & Stitt, L. (2019). Intravenous lidocaine in the management of chronic peripheral neuropathic pain: A randomized-controlled trial. *Canadian Journal of Anaesthesia = Journal Canadien D’anesthesie*, *66*(7), 820–827.<https://doi.org/10.1007/s12630-019-01395-8>
125. Mu, Y., Liu, X., Li, Q., Chen, K., Liu, Y., Lv, X., Xu, X., Fan, D., Shang, N., Yang, R., Pauer, L., & Pan, C. (2018). Efficacy and safety of pregabalin for painful diabetic peripheral neuropathy in a population of Chinese patients: A randomized placebo-controlled trial. *Journal of Diabetes*, *10*(3), 256–265.<https://doi.org/10.1111/1753-0407.12585>
126. Nalamachu, S., Hale, M., & Khan, A. (2014). Hydromorphone extended release for neuropathic and non-neuropathic/nociceptive chronic low back pain: A post hoc analysis of data from a randomized, multicenter, double-blind, placebo-controlled clinical trial. *Journal of Opioid Management*, *10*(5), 311–322.<https://doi.org/10.5055/jom.2014.0221>
127. Nardone, R., Höller, Y., Langthaler, P. B., Lochner, P., Golaszewski, S., Schwenker, K., Brigo, F., & Trinka, E. (2017). rTMS of the prefrontal cortex has analgesic effects on neuropathic pain in subjects with spinal cord injury. *Spinal Cord*, *55*(1), 20–25.<https://doi.org/10.1038/sc.2016.87>
128. Ngernyam, N., Jensen, M. P., Arayawichanon, P., Auvichayapat, N., Tiamkao, S., Janjarasjitt, S., Punjaruk, W., Amatachaya, A., Aree-uea, B., & Auvichayapat, P. (2015). The effects of transcranial direct current stimulation in patients with neuropathic pain from spinal cord injury. *Clinical Neurophysiology: Official Journal of the International Federation of Clinical Neurophysiology*, *126*(2), 382–390.<https://doi.org/10.1016/j.clinph.2014.05.034>
129. Onesti, E., Gabriele, M., Cambieri, C., Ceccanti, M., Raccah, R., Di Stefano, G., Biasiotta, A., Truini, A., Zangen, A., & Inghilleri, M. (2013). H ‐coil repetitive transcranial magnetic stimulation for pain relief in patients with diabetic neuropathy. *European Journal of Pain*, *17*(9), 1347–1356.<https://doi.org/10.1002/j.1532-2149.2013.00320.x>
130. Ostenfeld, T., Krishen, A., Lai, R. Y., Bullman, J., Baines, A. J., Green, J., Anand, P., & Kelly, M. (2013). Analgesic efficacy and safety of the novel p38 MAP kinase inhibitor, losmapimod, in patients with neuropathic pain following peripheral nerve injury: A double-blind, placebo-controlled study. *European Journal of Pain*, *17*(6), 844–857.<https://doi.org/10.1002/j.1532-2149.2012.00256.x>
131. Ostenfeld, T., Krishen, A., Lai, R. Y., Bullman, J., Green, J., Anand, P., Scholz, J., & Kelly, M. (2015). A randomized, placebo-controlled trial of the analgesic efficacy and safety of the p38 MAP kinase inhibitor, losmapimod, in patients with neuropathic pain from lumbosacral radiculopathy. *The Clinical Journal of Pain*, *31*(4), 283–293.<https://doi.org/10.1097/AJP.0000000000000122>
132. Otis, J. D., Sanderson, K., Hardway, C., Pincus, M., Tun, C., & Soumekh, S. (2013). A randomized controlled pilot study of a cognitive-behavioral therapy approach for painful diabetic peripheral neuropathy. *The Journal of Pain*, *14*(5), 475–482.<https://doi.org/10.1016/j.jpain.2012.12.013>
133. Özkul, Ç., Kılınç, M., Yıldırım, S. A., Topçuoğlu, E. Y., & Akyüz, M. (2015). Effects of visual illusion and transcutaneous electrical nerve stimulation on neuropathic pain in patients with spinal cord injury: A randomised controlled cross-over trial. *Journal of Back and Musculoskeletal Rehabilitation*, *28*(4), 709–719.<https://doi.org/10.3233/BMR-140573>
134. Palladini, M., Boesl, I., Koenig, S., Buchheister, B., & Attal, N. (2019). Lidocaine medicated plaster, an additional potential treatment option for localized post-surgical neuropathic pain: Efficacy and safety results of a randomized, placebo-controlled trial. *Current Medical Research and Opinion*, *35*(5), 757–766.<https://doi.org/10.1080/03007995.2019.1565709>
135. Parsons, B., Emir, B., & Knapp, L. (2012). Examining the time-to-improvement of sleep disturbance in patients with painful diabetic peripheral neuropathy and post-herpetic neuralgia. *The Journal of Pain*, *13*(4), S63.<https://doi.org/10.1016/j.jpain.2012.01.262>
136. Petersen, E. A., Stauss, T. G., Scowcroft, J. A., Brooks, E. S., White, J. L., Sills, S. M., Amirdelfan, K., Guirguis, M. N., Xu, J., Yu, C., Nairizi, A., Patterson, D. G., Tsoulfas, K. C., Creamer, M. J., Galan, V., Bundschu, R. H., Paul, C. A., Mehta, N. D., Choi, H., … Mekhail, N. A. (2021). Effect of High-frequency (10-kHz) Spinal Cord Stimulation in Patients With Painful Diabetic Neuropathy: A Randomized Clinical Trial. *JAMA Neurology*, *78*(6), 687–698.<https://doi.org/10.1001/jamaneurol.2021.0538>
137. Petramfar, P., Moein, M., Samani, S. M., Tabatabaei, S. H., & Zarshenas, M. M. (2016). Trachyspermum ammi 10 % topical cream versus placebo on neuropathic pain, a randomized, double-blind, placebo-controlled trial. *Neurological Sciences: Official Journal of the Italian Neurological Society and of the Italian Society of Clinical Neurophysiology*, *37*(9), 1449–1455.<https://doi.org/10.1007/s10072-016-2600-3>
138. Pickering, G., Pereira, B., Dufour, E., Soule, S., & Dubray, C. (2014). Impaired modulation of pain in patients with postherpetic neuralgia. *Pain Research & Management : The Journal of the Canadian Pain Society*, *19*(1), e19–e23.<https://doi.org/10.1155/2014/507947>
139. Pickering, G., Pereira, B., Morel, V., Corriger, A., Giron, F., Marcaillou, F., Bidar-Beauvallot, A., Chandeze, E., Lambert, C., Bernard, L., & Delage, N. (2020). Ketamine and Magnesium for Refractory Neuropathic Pain: A Randomized, Double-blind, Crossover Trial. *Anesthesiology*, *133*(1), 154–164.<https://doi.org/10.1097/ALN.0000000000003345>
140. Pickering, G., Voute, M., Macian, N., Ganry, H., & Pereira, B. (2019). Effectiveness and safety of 5% lidocaine-medicated plaster on localized neuropathic pain after knee surgery: A randomized, double-blind controlled trial. *Pain*, *160*(5), 1186–1195.<https://doi.org/10.1097/j.pain.0000000000001502>
141. Ponirakis, G., Abdul‐Ghani, M. A., Jayyousi, A., Zirie, M. A., Qazi, M., Almuhannadi, H., Petropoulos, I. N., Khan, A., Gad, H., Migahid, O., Megahed, A., Al‐Mohannadi, S., AlMarri, F., Al‐Khayat, F., Mahfoud, Z., Al Hamad, H., Ramadan, M., DeFronzo, R., & Malik, R. A. (2021). Painful diabetic neuropathy is associated with increased nerve regeneration in patients with type 2 diabetes undergoing intensive glycemic control. *Journal of Diabetes Investigation*, *12*(9), 1642–1650.<https://doi.org/10.1111/jdi.13544>
142. Pozeg, P., Palluel, E., Ronchi, R., Solcà, M., Al-Khodairy, A.-W., Jordan, X., Kassouha, A., & Blanke, O. (2017). Virtual reality improves embodiment and neuropathic pain caused by spinal cord injury. *Neurology*, *89*(18), 1894–1903.<https://doi.org/10.1212/WNL.0000000000004585>
143. Quiding, H., Åkermark, C., Segerdahl, M., Reinholdsson, I., Svensson, H., & Jonzon, B. (2013). A pain model with a neuropathic somatosensory lesion: Morton neuroma. *Pain*, *154*(11), 2494–2499.<https://doi.org/10.1016/j.pain.2013.07.030>
144. Radic, J. A. E., Beauprie, I., Chiasson, P., Kiss, Z. H. T., & Brownstone, R. M. (2015). Motor Cortex Stimulation for Neuropathic Pain: A Randomized Cross-over Trial. *The Canadian Journal of Neurological Sciences. Le Journal Canadien Des Sciences Neurologiques*, *42*(6), 401–409.<https://doi.org/10.1017/cjn.2015.292>
145. Raptis, E., Vadalouca, A., Stavropoulou, E., Argyra, E., Melemeni, A., & Siafaka, I. (2014). Pregabalin vs. opioids for the treatment of neuropathic cancer pain: A prospective, head-to-head, randomized, open-label study. *Pain Practice: The Official Journal of World Institute of Pain*, *14*(1), 32–42.<https://doi.org/10.1111/papr.12045>
146. Raskin, P., Huffman, C., Toth, C., Asmus, M. J., Messig, M., Sanchez, R. J., & Pauer, L. (2014). Pregabalin in patients with inadequately treated painful diabetic peripheral neuropathy: A randomized withdrawal trial. *The Clinical Journal of Pain*, *30*(5), 379–390.<https://doi.org/10.1097/AJP.0b013e31829ea1a1>
147. Raskin, P., Huffman, C., Yurkewicz, L., Pauer, L., Scavone, J. M., Yang, R., & Parsons, B. (2016). Pregabalin in Patients With Painful Diabetic Peripheral Neuropathy Using an NSAID for Other Pain Conditions: A Double-Blind Crossover Study. *The Clinical Journal of Pain*, *32*(3), 203–210.<https://doi.org/10.1097/AJP.0000000000000254>
148. Rastogi, A., Uppula, P., Saikia, U., & Bhansali, A. (2021). Effect of Monochromatic Infrared Energy on Quality of Life and Intraepidermal Nerve Fiber Density in Painful Diabetic Neuropathy: A Randomized, Sham Control Study. *Neurology India*, *69*(5), 1331–1337.<https://doi.org/10.4103/0028-3886.329614>
149. Rauck, R., Makumi, C. W., Schwartz, S., Graff, O., Meno-Tetang, G., Bell, C. F., Kavanagh, S. T., & McClung, C. L. (2013). A randomized, controlled trial of gabapentin enacarbil in subjects with neuropathic pain associated with diabetic peripheral neuropathy. *Pain Practice: The Official Journal of World Institute of Pain*, *13*(6), 485–496.<https://doi.org/10.1111/papr.12014>
150. Razak, I., Chung, T. Y., & Ahmad, T. S. (2019). A Comparative Study of Two Modalities in Pain Management of Patients Presenting with Chronic Brachial Neuralgia. *Journal of Alternative and Complementary Medicine*, *25*(8), 861–867.<https://doi.org/10.1089/acm.2019.0052>
151. Razazian, N., Baziyar, M., Moradian, N., Afshari, D., Bostani, A., & Mahmoodi, M. (2014). Evaluation of the efficacy and safety of pregabalin, venlafaxine, and carbamazepine in patients with painful diabetic peripheral neuropathy. A randomized, double-blind trial. *Neurosciences*, *19*(3), 192–198.
152. Rigo, F. K., Trevisan, G., Godoy, M. C., Rossato, M. F., Dalmolin, G. D., Silva, M. A., Menezes, M. S., Caumo, W., & Ferreira, J. (2017). Management of Neuropathic Chronic Pain with Methadone Combined with Ketamine: A Randomized, Double Blind, Active-Controlled Clinical Trial. *Pain Physician*, *20*(3), 207–215.
153. Rozworska, K. A., Poulin, P. A., Carson, A., Tasca, G. A., & Nathan, H. J. (2020). Mediators and moderators of change in mindfulness-based stress reduction for painful diabetic peripheral neuropathy. *Journal of Behavioral Medicine*, *43*(2), 297–307.<https://doi.org/10.1007/s10865-019-00079-4>
154. Sandoval, R., Roddey, T., Giordano, T. P., Mitchell, K., & Kelley, C. (2016). Randomized Trial of Lower Extremity Splinting to Manage Neuropathic Pain and Sleep Disturbances in People Living with HIV/AIDS. *Journal of the International Association of Providers of AIDS Care*, *15*(3), 240–247.<https://doi.org/10.1177/2325957413511112>
155. Sang, C. N., Sathyanarayana, R., Sweeney, M., & DM-1796 Study Investigators. (2013). Gastroretentive gabapentin (G-GR) formulation reduces intensity of pain associated with postherpetic neuralgia (PHN). *The Clinical Journal of Pain*, *29*(4), 281–288.<https://doi.org/10.1097/AJP.0b013e318258993e>
156. Sansone, P., Passavanti, M. B., Fiorelli, A., Aurilio, C., Colella, U., De Nardis, L., Donatiello, V., Pota, V., & Pace, M. C. (2017). Efficacy of the topical 5% lidocaine medicated plaster in the treatment of chronic post-thoracotomy neuropathic pain. *Pain Management*, *7*(3), 189–196.<https://doi.org/10.2217/pmt-2016-0060>
157. Sarısoy, P., & Ovayolu, O. (2020). The Effect of Foot Massage on Peripheral Neuropathy-Related Pain and Sleep Quality in Patients With Non-Hodgkin’s Lymphoma. *Holistic Nursing Practice*, *34*(6), 345–355.<https://doi.org/10.1097/HNP.0000000000000412>
158. Schimrigk, S., Marziniak, M., Neubauer, C., Kugler, E. M., Werner, G., & Abramov-Sommariva, D. (2017). Dronabinol Is a Safe Long-Term Treatment Option for Neuropathic Pain Patients. *European Neurology*, *78*(5–6), 320–329.<https://doi.org/10.1159/000481089>
159. Schug, S. A., Parsons, B., Almas, M., & Whalen, E. (2017). Effect of Concomitant Pain Medications on Response to Pregabalin in Patients with Postherpetic Neuralgia or Spinal Cord Injury-Related Neuropathic Pain. *Pain Physician*, *20*(1), E53–E63.
160. Schukro, R. P., Oehmke, M. J., Geroldinger, A., Heinze, G., Kress, H.-G., & Pramhas, S. (2016). Efficacy of Duloxetine in Chronic Low Back Pain with a Neuropathic Component: A Randomized, Double-blind, Placebo-controlled Crossover Trial. *Anesthesiology*, *124*(1), 150–158.<https://doi.org/10.1097/ALN.0000000000000902>
161. Serpell, M., Ratcliffe, S., Hovorka, J., Schofield, M., Taylor, L., Lauder, H., & Ehler, E. (2014). A double-blind, randomized, placebo-controlled, parallel group study of THC/CBD spray in peripheral neuropathic pain treatment. *European Journal of Pain*, *18*(7), 999–1012.<https://doi.org/10.1002/j.1532-2149.2013.00445.x>
162. Serra, J., Duan, W. R., Locke, C., Solà, R., Liu, W., & Nothaft, W. (2015). Effects of a T-type calcium channel blocker, ABT-639, on spontaneous activity in C-nociceptors in patients with painful diabetic neuropathy: A randomized controlled trial. *Pain*, *156*(11), 2175–2183.<https://doi.org/10.1097/j.pain.0000000000000249>
163. Shergill, Y., Rice, D. B., Khoo, E.-L., Jarvis, V., Zhang, T., Taljaard, M., Wilson, K. G., Romanow, H., Glynn, B., Small, R., Rash, J. A., Smith, A., Monteiro, L., Smyth, C., & Poulin, P. A. (2022). Mindfulness-Based Stress Reduction in Breast Cancer Survivors with Chronic Neuropathic Pain: A Randomized Controlled Trial. *Pain Research & Management*, *2022*, 4020550.<https://doi.org/10.1155/2022/4020550>
164. Shimizu, T., Hosomi, K., Maruo, T., Goto, Y., Yokoe, M., Kageyama, Y., Shimokawa, T., Yoshimine, T., & Saitoh, Y. (2017). Efficacy of deep rTMS for neuropathic pain in the lower limb: A randomized, double-blind crossover trial of an H-coil and figure-8 coil. *Journal of Neurosurgery*, *127*(5), 1172–1180.<https://doi.org/10.3171/2016.9.JNS16815>
165. Shokri, M., Sajedi, F., Mohammadi, Y., & Mehrpooya, M. (2021). Adjuvant use of melatonin for relieving symptoms of painful diabetic neuropathy: Results of a randomized, double-blinded, controlled trial. *European Journal of Clinical Pharmacology*, *77*(11), 1649–1663.<https://doi.org/10.1007/s00228-021-03170-5>
166. Simpson, D. M., Rice, A. S. C., Emir, B., Landen, J., Semel, D., Chew, M. L., & Sporn, J. (2014). A randomized, double-blind, placebo-controlled trial and open-label extension study to evaluate the efficacy and safety of pregabalin in the treatment of neuropathic pain associated with human immunodeficiency virus neuropathy. *Pain*, *155*(10), 1943–1954.<https://doi.org/10.1016/j.pain.2014.05.027>
167. Simpson, R. W., & Wlodarczyk, J. H. (2016). Transdermal Buprenorphine Relieves Neuropathic Pain: A Randomized, Double-Blind, Parallel-Group, Placebo-Controlled Trial in Diabetic Peripheral Neuropathic Pain. *Diabetes Care*, *39*(9), 1493–1500.<https://doi.org/10.2337/dc16-0123>
168. Slangen, R., Schaper, N. C., Faber, C. G., Joosten, E. A., Dirksen, C. D., van Dongen, R. T., Kessels, A. G., & van Kleef, M. (2014). Spinal cord stimulation and pain relief in painful diabetic peripheral neuropathy: A prospective two-center randomized controlled trial. *Diabetes Care*, *37*(11), 3016–3024.<https://doi.org/10.2337/dc14-0684>
169. Smith, E. M. L., Pang, H., Cirrincione, C., Fleishman, S., Paskett, E. D., Ahles, T., Bressler, L. R., Fadul, C. E., Knox, C., Le-Lindqwister, N., Gilman, P. B., Shapiro, C. L., & Alliance for Clinical Trials in Oncology. (2013). Effect of duloxetine on pain, function, and quality of life among patients with chemotherapy-induced painful peripheral neuropathy: A randomized clinical trial. *JAMA*, *309*(13), 1359–1367.<https://doi.org/10.1001/jama.2013.2813>
170. Srinivasan, A., Dutta, P., Bansal, D., Chakrabarti, A., Bhansali, A. K., & Hota, D. (2021). Efficacy and safety of low-dose naltrexone in painful diabetic neuropathy: A randomized, double-blind, active-control, crossover clinical trial. *Journal of Diabetes*, *13*(10), 770–778.<https://doi.org/10.1111/1753-0407.13202>
171. Tanenberg, R. J., Clemow, D. B., Giaconia, J. M., & Risser, R. C. (2014). Duloxetine Compared with Pregabalin for Diabetic Peripheral Neuropathic Pain Management in Patients with Suboptimal Pain Response to Gabapentin and Treated with or without Antidepressants: A Post Hoc Analysis. *Pain Practice: The Official Journal of World Institute of Pain*, *14*(7), 640–648.<https://doi.org/10.1111/papr.12121>
172. Tarullo, Angelo, Tarullo, Antonella, Lacatena, E., Santacroce, L., Inchingolo, A. M., Malcangi, G., Scacco, S., Fanelli, M., Cagiano, R., Inchingolo, F., Caprio, M., Dipalma, G., Ballini, A., Clemente, A., Inchingolo, A. D., Girolamo, F., & Tattoli, M. (2019). Topical Fisionerv® is effective in treatment of peripheral neuropathic pain. *Acta Bio-Medica: Atenei Parmensis*, *90*(1), 51–55.<https://doi.org/10.23750/abm.v90i1.6305>
173. Tesfaye, S., Sloan, G., Petrie, J., White, D., Bradburn, M., Young, T., Rajbhandari, S., Sharma, S., Rayman, G., Gouni, R., Alam, U., Julious, S. A., Cooper, C., Loban, A., Sutherland, K., Glover, R., Waterhouse, S., Turton, E., Horspool, M., … Selvarajah, D. (2022). Optimal pharmacotherapy pathway in adults with diabetic peripheral neuropathic pain: The OPTION-DM RCT. *Health Technology Assessment*, *26*(39), 1–100.<https://doi.org/10.3310/RXUO6757>
174. Tesfaye, S., Wilhelm, S., Lledo, A., Schacht, A., Tölle, T., Bouhassira, D., Cruccu, G., Skljarevski, V., & Freynhagen, R. (2013). Duloxetine and pregabalin: High-dose monotherapy or their combination? The “COMBO-DN study”--a multinational, randomized, double-blind, parallel-group study in patients with diabetic peripheral neuropathic pain. *Pain*, *154*(12), 2616–2625.<https://doi.org/10.1016/j.pain.2013.05.043>
175. Tiendrebeogo, E., Choueiri, M., Chevalier, X., Conrozier, T., & Eymard, F. (2021). Does the Presence of Neuropathic Pain Influence the Response to Hyaluronic Acid in Patients with Knee Osteoarthritis? *Cartilage*, *13*(1_suppl), 1548S-1556S.<https://doi.org/10.1177/1947603520954509>
176. Toth, C., Brady, S., Gagnon, F., & Wigglesworth, K. (2014). A randomized, single-blind, controlled, parallel assignment study of exercise versus education as adjuvant in the treatment of peripheral neuropathic pain. *The Clinical Journal of Pain*, *30*(2), 111–118.<https://doi.org/10.1097/AJP.0b013e31828ccd0f>
