# Supplementary figures and images for: Exclusion criteria in clinical trials of treatments for neuropathic pain: a systematic analysis
Source: Front Pain Res (Lausanne). 2026 Mar 18;7:1716686. doi: 10.3389/fpain.2026.1716686 (PMC13038933; doi:10.3389/fpain.2026.1716686)

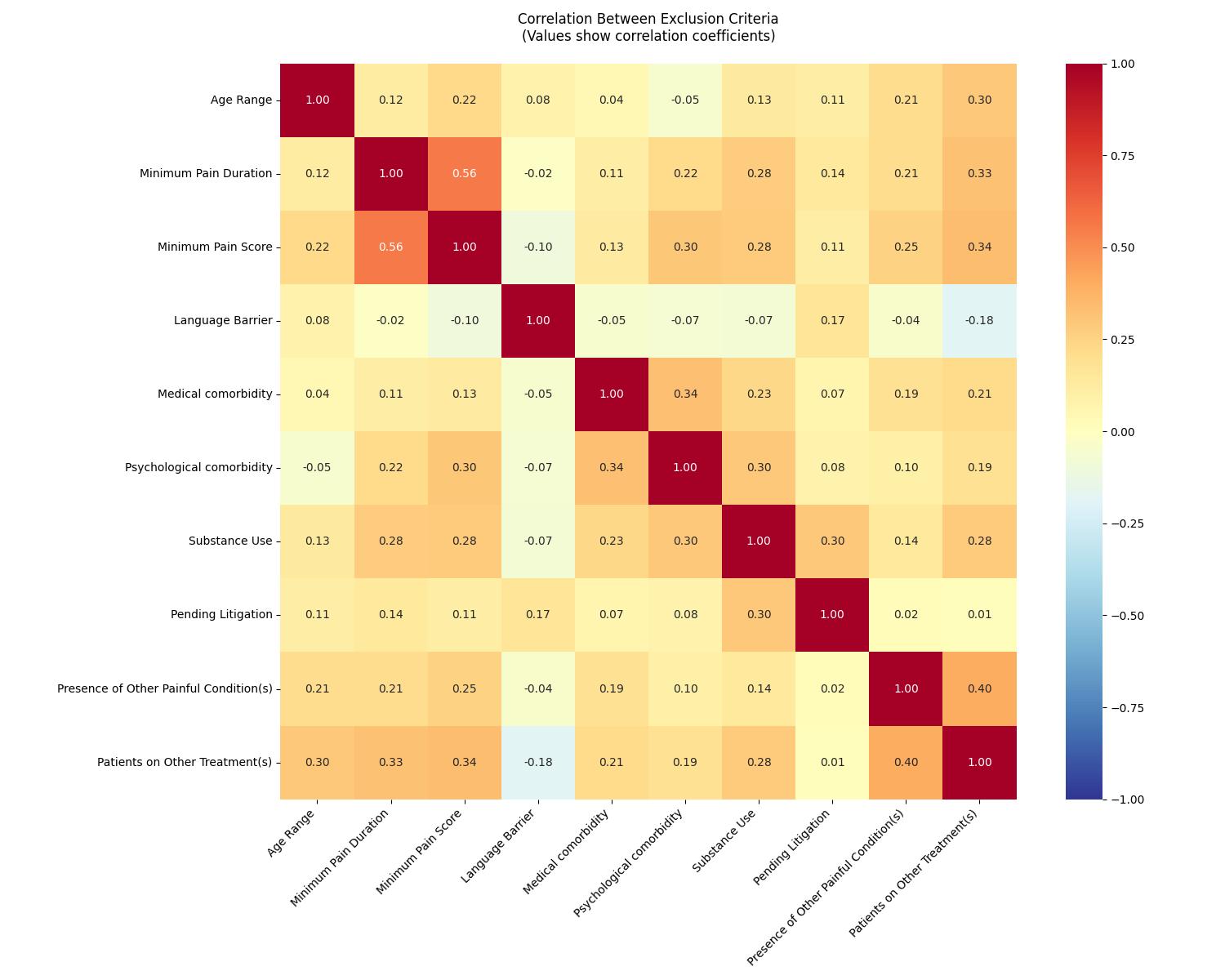

Supplement: Supplementary file 2 [file Image1.jpeg]
